# Glycosylation is an Androgen-Regulated Process Essential for Prostate Cancer Cell Viability^☆^

**DOI:** 10.1016/j.ebiom.2016.04.018

**Published:** 2016-04-20

**Authors:** Jennifer Munkley, Daniel Vodak, Karen E. Livermore, Katherine James, Brian T. Wilson, Bridget Knight, Paul Mccullagh, John Mcgrath, Malcolm Crundwell, Lorna W. Harries, Hing Y. Leung, Craig N. Robson, Ian G. Mills, Prabhakar Rajan, David J. Elliott

**Affiliations:** a Institute of Genetic Medicine, Newcastle University, Newcastle upon Tyne NE1 3BZ, UK; b Bioinformatics Core Facility, Institute for Cancer Genetics and Informatics, The Norwegian Radium Hospital, Oslo University Hospital, Oslo, Norway; c Interdisciplinary Computing and Complex BioSystems Research Group, Newcastle University, Newcastle upon Tyne, UK; d Northern Genetics Service, Newcastle Upon Tyne NHS Foundation Trust, International Centre for Life, Newcastle upon Tyne, UK; e NIHR Exeter Clinical Research Facility, RD&E NHS Foundation Trust, UK; f Department of Pathology, RD&E NHS Foundation Trust, UK; g Exeter Surgical Health Services Research Unit, RD&E NHS Foundation Trust, UK; h Department of Urology, Royal Devon and Exeter NHS Foundation Trust, Exeter, UK; i Institute of Biomedical and Clinical Sciences, University of Exeter, Devon EX1 2LU, UK; j Cancer Research UK Beatson Institute, Glasgow G61 1BD, UK; k Institute of Cancer Sciences, University of Glasgow, Glasgow G12 8QQ, UK; l Northern Institute for Cancer Research, Newcastle University, Newcastle upon Tyne NE2 4HH, UK; m Prostate Cancer Research Group, Centre for Molecular Medicine Norway (NCMM), University of Oslo and Oslo University Hospitals, Oslo, Norway; n Departments of Molecular Oncology, Institute of Cancer Research and Radium Hospital, Oslo, Norway; o PCUK/Movember Centre of Excellence for Prostate Cancer Research, Centre for Cancer Research and Cell Biology (CCRCB), Queen's University, Belfast, UK; p Barts Cancer Institute, Queen Mary University of London, John Vane Science Centre, Charterhouse Square, London EC1M 6BQ, UK

## Abstract

Steroid androgen hormones play a key role in the progression and treatment of prostate cancer, with androgen deprivation therapy being the first-line treatment used to control cancer growth. Here we apply a novel search strategy to identify androgen-regulated cellular pathways that may be clinically important in prostate cancer. Using RNASeq data, we searched for genes that showed reciprocal changes in expression in response to acute androgen stimulation in culture, and androgen deprivation in patients with prostate cancer. Amongst 700 genes displaying reciprocal expression patterns we observed a significant enrichment in the cellular process glycosylation. Of 31 reciprocally-regulated glycosylation enzymes, a set of 8 (*GALNT7*, *ST6GalNAc1*, *GCNT1*, *UAP1*, *PGM3*, *CSGALNACT1*, *ST6GAL1* and EDEM3) were significantly up-regulated in clinical prostate carcinoma. Androgen exposure stimulated synthesis of glycan structures downstream of this core set of regulated enzymes including sialyl-Tn (sTn), sialyl Lewis^X^ (SLe^X^), *O*-GlcNAc and chondroitin sulphate, suggesting androgen regulation of the core set of enzymes controls key steps in glycan synthesis. Expression of each of these enzymes also contributed to prostate cancer cell viability. This study identifies glycosylation as a global target for androgen control, and suggests loss of specific glycosylation enzymes might contribute to tumour regression following androgen depletion therapy.

## Highlights

• RNA-sequencing of a prostate cancer cell line and 7 prostate cancer patients identifies a core set of 700 clinically relevant androgen-regulated genes.
• Glycosylation is a global target for androgen control in prostate cancer cells.
• A set of 8 glycosylation enzymes are important for prostate cancer cell viability.

## 1 Introduction

Prostate cancer (PCa) is the second most common cause of male cancer death in the United States, and there is an urgent need to identify the key mechanisms that drive clinical disease progression and its response to treatment, biomarkers for disease progression and therapeutic targets. Male steroid hormones called androgens play a critical role in PCa progression, and androgen deprivation therapy (ADT) is the first line treatment for PCa. Although ADT is usually initially effective, many patients ultimately develop lethal castrate-resistant PCa (CRPCa) for which treatment options are limited. Androgens control gene expression via a transcription factor called the androgen receptor (AR). The AR is essential for PCa cell viability, proliferation and invasion (Snoek et al., 2009, Hara et al., 2008, Haag et al., 2005), and in CRPCa there is a strong selective pressure on cells to maintain AR-regulated signalling pathways even in lower conditions of circulating androgens. Progression to CRPCa is thought to involve persistence of AR signalling and reprogramming of the AR transcriptional landscape (Sharma et al., 2013, Mills, 2014), during which time AR activity is maintained through activating mutations (Veldscholte et al., 1990, Steinkamp et al., 2009), gene amplification (Visakorpi et al., 1995), AR splice variants (Sun et al., 2010), or signalling crosstalk with other oncogenic pathways (Craft et al., 1999).

Classically, androgen binding is thought to promote dimerization of AR and its translocation to the nucleus where it can act as either a transcriptional enhancer or repressor, depending on the target gene (Karantanos et al., 2013, Cai et al., 2011). Various genomic and transcriptomic approaches have been used to identify both AR binding sites and target genes. However, these studies have generally focused either on the use of a single model of prostate cancer (LNCaP or its in vitro derived subclones) (Massie et al., 2007, Wang et al., 2007, Rajan et al., 2011), only on AR regulated genes with nearby AR genomic binding sites (Massie et al., 2011, Sharma et al., 2013), or have been limited by genome coverage on microarrays (Rajan et al., 2011, Massie et al., 2007, Massie et al., 2011, Wang et al., 2009a). Genome-wide mapping of AR binding was recently achieved in prostate tissue using chromatin immunoprecipitation sequencing (ChIP-seq) (Sharma et al., 2013). This study successfully revealed an in vivo AR regulated transcriptional program in CRPCa (Sharma et al., 2013), but was limited by the challenges of directly associating an AR binding site with a given gene and its expression (Barfeld et al., 2014, Wu et al., 2011).

Recent RNA—Seq analysis of metastatic CRPCa revealed that the majority of metastatic PCa harbours clinically actionable molecular alterations (Robinson et al., 2015), providing hope for precision medicine in affected individuals. However, despite this finding, the mechanisms through which androgens drive the initial development and growth of PCa remain poorly understood. To address these issues we used RNA-Seq to comprehensively profile how the PCa transcriptome responds to androgens. Since previous studies have demonstrated the importance of cellular context in the functional positioning of the AR (Sharma et al., 2013), we directly correlated this gene expression data after androgen stimulation with RNA-Seq data from 7 PCa patients before and after receiving ADT (Rajan et al., 2014). This combined approach identified a core set of nearly 700 genes which were reciprocally regulated between the two datasets and so are strong candidates for being clinically relevant androgen-regulated genes within localised cancers. Gene-set enrichment of these 700 genes revealed a multi-factorial link between androgens and the PCa glycoproteome, and specifically a core group of 7 glycosylation enzymes that significantly change expression in clinical PCa. Importantly, within this core set, 6 enzymes have not previously been implicated in PCa. Our data suggest that androgen regulation of these 8 core enzymes may be rate limiting for the expression of cancer-associated glycans and suggests loss of glycosylation enzymes might contribute to tumour regression following ADT.

## 2 Materials and Methods

### 2.1 RNA-Seq and Analysis

For RNA-Seq of LNCaP cells: RNA was prepared for sequencing using a RNA Easy Kit (Qiagen 74104). All RNA samples were DNase I treated using a DNA-free kit (Invitrogen) and stored at − 80 °C prior to RNA quality control check using 2100 Agilent Bioanalyser and mRNA library prep using TruSeq mRNA library kit (Illumina). Pair-end sequencing was done in total for six samples (three biological replicates of LNCaP cells grown in charcoal stripped steroid deplete media, and three biological replicates from LNCaP cells grown in the presence of 10 nM R1881 for 24 h) using an Illumina HiSeq 2000. RNA-Seq of RNA from 7 patients before and after ADT (Clinical dataset A) was as described previously (Rajan et al., 2014). Expression distribution, scatter and volcano polts for both RNA-Seq datasets are shown in Supplementary Figs. 2a & 2b.

The RNA-Seq analysis of both datasets was carried out as follows: Differential gene and transcript expression analysis was performed according to the Tuxedo protocol (Trapnell et al., 2012). Separately for each of the clinical (7 patients pre- and post ADT, clinical cohort A), and cell-line samples (LNCaP cells grown with or without androgens), all reads were first mapped to human transcriptome/genome (build hg19) with TopHat (Kim et al., 2013) /Bowtie (Langmead and Salzberg, 2012), followed by per-sample transcript assembly with Cufflinks (Trapnell et al., 2013). Transcript assemblies derived from the clinical samples were merged together with Cuffmerge (Trapnell et al., 2013), and differential expression between the two conditions (prior and post androgen-deprivation therapy) was assessed with Cuffdiff (Trapnell et al., 2013). Genes/isoforms labelled by the software as significantly differentially expressed were extracted from the results. The mapped LNCaP data was processed with the same pipeline (with Cuffmerge, Cuffdiff and Cuffcompare, followed by extraction of significantly differentially expressed genes/isoforms); in this case, expression changes between cells grown with or without androgens were assessed. Two subsets of significantly differentially expressed genes were identified: 1) Genes up-regulated by androgens in LNCaP cells and down-regulated in 7 patients following androgen-deprivation therapy. 2) Genes down-regulated by androgens in LNCaP cells and up-regulated in 7 patients following androgen-deprivation therapy.

### 2.2 Gene Ontology Analysis

GO analysis was carried out as described previously (Young et al., 2010). Enrichment of GO biological processes (with < 500 annotations) was calculated using the goseq R package (version 1.18.0). Genes were considered significant at a *p*-value threshold of 0.05 after adjustment using the Benjamini-Hochberg false discovery rate.

### 2.3 Antibodies

The antibodies and lectins used in the study are listed in Supplementary Table 1. The specificity of all of the antibodies used was validated by siRNA mediated protein depletion (Supplementary Fig. 2).

### 2.4 Cell culture

Cell culture and the cell lines used were as described previously (Munkley et al., 2014, Munkley et al., 2015a, Munkley et al., 2015b, Munkley et al., 2015c). All siRNA sequences are listed in Supplementary Table 1.

### 2.5 Clinical Samples

Clinical dataset A: The patient samples used for RNA-Sequencing were as described previously (Rajan et al., 2014). RNA was obtained from seven patients with locally advanced metastatic prostate cancer before and ~ 22 weeks after ADT. The clinical samples used in clinical datasets B and C are as described previously (Munkley et al., 2015b, Munkley et al., 2015c). The samples were obtained with ethical approval through the Exeter NIHR Clinical Research Facility tissue bank (Ref: STB20). Written informed consent for the use of surgically obtained tissue was provided by all patients.

### 2.6 RT-qPCR

RT-qPCR was carried out as described previously (Munkley et al., 2015c). All primer sequences are listed in Supplementary Table 1.

### 2.7 Cell Viability Analysis

Cell viability analysis was carried out using the TaliR Cell Viability Kit (Life Technologies A10786) and the TaliR Image-based Cytometer. Relative cell numbers following siRNA treatment were determined using the TaliR Image-based Cytometer (Life Technologies).

## 3 Results

### 3.1 Reciprocal Gene Expression Patterns Identify a Core Set of Clinically Relevant Androgen-Regulated Target Genes

**Fig. 1:**
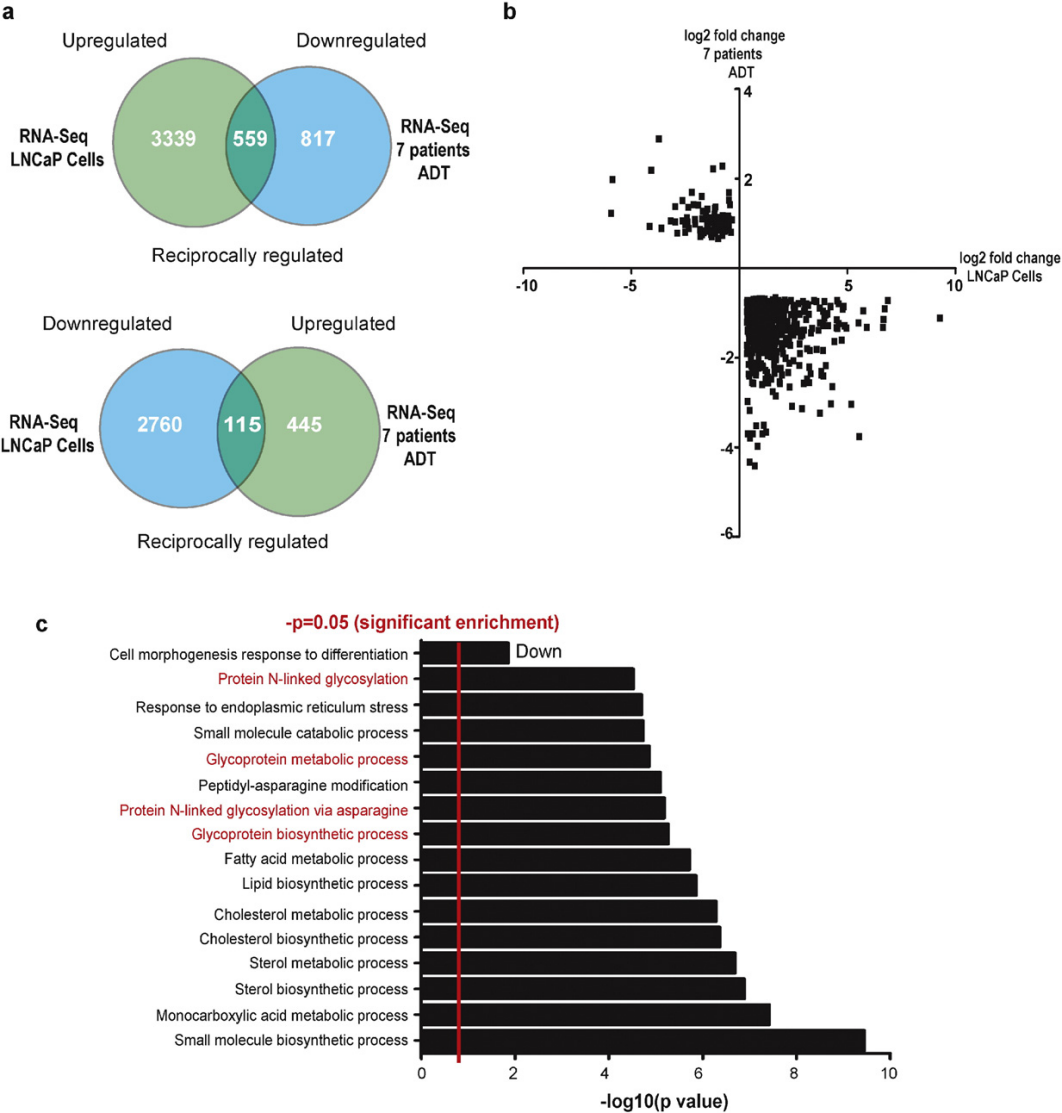
Reciprocal gene expression signatures identify a core set of clinically relevant androgen-regulated target genes. (A) RNA-Seq expression analysis of LNCaP cells treated with or without 10 nM R1881 (a synthetic androgen) for 24 h in triplicate, and of 7 PCa patient prostate biopsies before and after androgen ablation therapy (ADT). Venn diagrams show the number of genes with significant differential expression (q < 0.05). Comparison of the two RNA-Seq datasets identified approximately 700 androgen-regulated genes with reciprocal regulation. 559 genes were up-regulated by androgen treatment in LNCaP cells, but down-regulated in PCa patient samples following ADT (upper Venn diagram). Conversely, 115 genes were down-regulated by androgen addition in LNCaP cells, but up-regulated in PCa patient samples following ADT (lower Venn diagram). (B) Scatter plot of reciprocally regulated genes between the two datasets. The X coordinates are expression log2 fold change values of individual genes in LNCaP cells treated with androgens, relative to cells grown in steroid deplete media. The Y coordinates are log2 fold changes in gene expression of individual genes in the patient dataset after ADT, relative to expression values before treatment. The log2 fold changes were calculated using the relative FPKM values for each gene. (C) Gene Ontology (GO) analysis of the reciprocally regulated genes identified 72 terms with significant gene enrichment (*p* < 0.05). The top 15 significantly enriched terms are shown in the graph.

To analyse how the PCa transcriptome globally responds to androgens we carried out triplicate RNA-Seq of LNCaP cells grown with or without 10 nM R1881 (a synthetic androgen) in vitro for 24 h. This procedure identified 3339 genes which were significantly up-regulated by androgens, and 2760 genes which were significantly down-regulated (q < 0.05) (Supplementary Tables 2 & 3). Since previous studies have demonstrated a divergence between in vivo and in vitro AR-regulated genes (Sharma et al., 2013), we correlated our cell line data with RNA-Seq data from 7 PCa patients before and after ADT(clinical dataset A) (Rajan et al., 2014). This process identified nearly 700 potentially clinically relevant AR target genes that showed reciprocal expression signatures, indicating that they are both androgen regulated in culture and have reverse expression switches in patients in response to clinical ADT (Fig. 1A, B).

### 3.2 Glycosylation is a Key Cellular Process Regulated by the AR

This comprehensive map of AR-regulated genes in clinical prostate cancer provides a new window through which to understand of the essential signalling pathways downstream of the AR and of the role these pathways play in both the development of PCa and the response to ADT. Within our dataset of 700 reciprocally regulated genes were many established classes of AR target genes, including cell cycle regulators (e.g. *ORC5L*), signalling molecules implicated in PCa (e.g. *HER3*), and genes with roles in central metabolism and biosynthetic pathways (e.g. *CAMKK2*), as well as many additional novel androgen-regulated genes (Supplementary Tables 4 & 5). Gene Ontology (GO) analysis of these 700 genes identified known AR regulated processes including lipid and cholesterol biosynthesis (Wu et al., 2014, Suburu and Chen, 2012, Swinnen et al., 1997b), fatty acid metabolism (Swinnen et al., 1997a, Swinnen et al., 2000, Liu, 2006), and response to ER stress (Segawa et al., 2002, Sheng et al., 2015) (Supplementary Table 6). Also, in addition to these previously known androgen-regulated cellular processes, ‘glycosylation’ was specifically identified as a previously unidentified androgen-regulated process, with a significant enrichment of genes encoding glycosylation enzymes within the dataset (Fig. 1C).

**Fig. 2:**
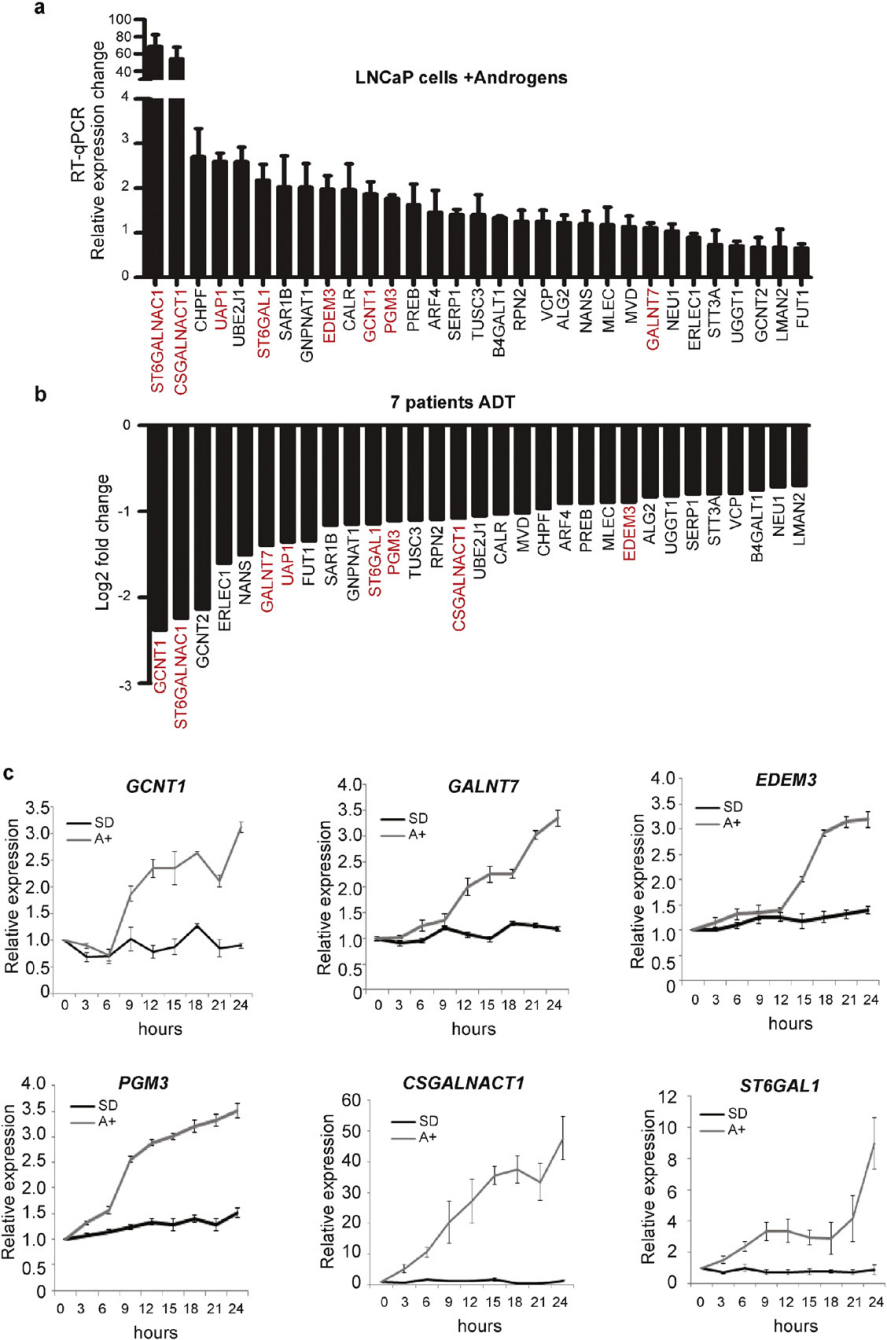
Thirty one glycosylation enzymes are regulated by androgens in PCa cells. The term ‘glycosylation’ was contained in 7 significantly enriched GO terms. (A) Real-time PCR validation of 31 androgen-regulated glycosylation related genes identified by GO analyses. LNCaP cells were grown in triplicate either with or without 10 nM R1881 (androgens) for 24 h. The Y axis shows the relative expression changes for each gene with androgens. These 31 glycosylation enzymes were significantly down-regulated in 7 PCa patients following ADT (B). The Y axis shows the relative expression level for each gene (calculated by comparing the FPKM expression values of genes after ADT to the value before treatment). Our meta-analysis of clinical PCa gene expression datasets identified 8 glycosylation enzymes, which were significantly (*p* < 0.05) upregulated in PCa relative to normal prostate tissue (Supplementary Table 7). (C) Real-time PCR analysis of glycosylation genes in LNCaP cells treated with or without androgens (10 nM R1881) over a 24 h period.

As mounting evidence links changes in the glycan composition of PCa cells to disease progression (Chen et al., 2014, Itkonen and Mills, 2013, Itkonen et al., 2014, Munkley et al., 2015c, Munkley et al., 2016), we further analysed our RNA—Seq data to identify 31 individual genes encoding glycosylation enzymes that display reciprocal gene expression between androgen stimulation in vitro, and ADT in patients (Fig. 2A, B). Meta-analysis of publically available clinical gene expression data across 326 samples in 3 large datasets (Taylor et al., 2010, Varambally et al., 2005, Grasso et al., 2012), and into two independent clinical RNA datasets, further resolved a subset of glycosylation genes that are additionally significantly up-regulated in PCa relative to the normal prostate gland (*p* < 0.05) (Supplementary Table 7). These genes are shown in red in Fig. 2A, B. Of these genes, *UAP1* and *ST6GalNAc1* are known AR target genes that have previously been linked with PCa progression (Itkonen et al., 2014, Munkley et al., 2015c, Munkley and Elliott, 2016b). However, to the best of our knowledge, this study is the first time that androgen-regulation of the other 6 genes (*GCNT1*, *GALNT7*, *PGM3*, *CSGALNACT1*, *ST6GAL1*, and *EDEM3*) has been described. Each of these 6 newly identified AR-regulated glycosylation enzyme genes showed a rapid pattern of activation following androgen stimulation in culture, suggesting they are early targets of the AR (Fig. 2C). Consistent with them being physiological targets of the AR, transcription of these genes was also activated by a range of R1881 concentrations, and inhibited by the AR antagonist Casodex® (Supplementary Fig. 3). We also confirmed androgen-regulation of all 8 glycosylation enzymes in the androgen-responsive VCaP prostate cancer cell line (Supplementary Fig. 4).

**Fig. 3:**
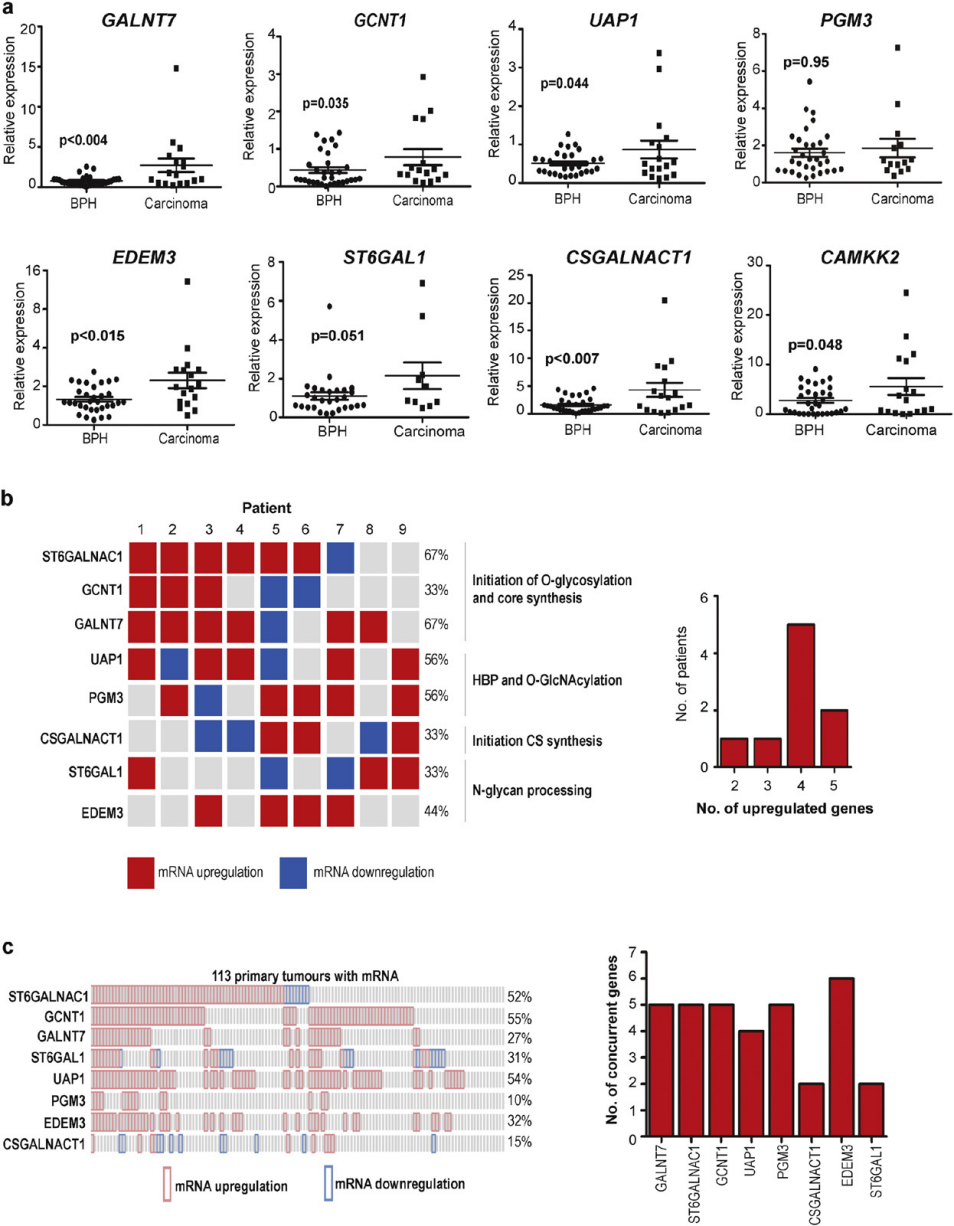
Core glycosylation gene signatures in clinical PCa RNA datasets. Meta-analysis of clinical RNA datasets identified 8 glycosylation enzymes with significant upregulation in PCa tissue (Supplementary Table 7). (A) Real-time PCR analysis of the expression of *UAP1*, *GCNT1*, *GALNT7*, *ST6GAL1*, *PGM3*, *EDEM3* and *CSGALNACT1* in an independent clinical RNA dataset (BPH v carcinoma, clinical cohort B). Expression of *ST6GALNAC1* mRNA was analysed in these samples previously (Munkley et al., 2015c). The CAMKK2 gene was used as a control. (B) Real-time PCR analysis of the expression of all 8 glycosylation enzymes in matched normal and tumour tissue samples from 9 patients (clinical cohort C) to monitor expression of individual genes in parallel in individual patients. Upregulated genes are shown in red, downregulated genes in blue, and genes not changing expression in grey (a significant gene expression change is recorded as > 1.6 fold change relative to 3 reference genes). Grey indicates there was no significant change in gene expression in PCa relative to a matched normal control. Red indicates significant mRNA upregulation, and blue indicates significant mRNA downregulation. The bar chart to the right shows that most patients show a pattern where more than one of the core glycosylation genes increase expression, with typically 4 genes changing expression concurrently. (C) Analysis of glycosylation enzyme mRNA expression in 113 primary prostate cancer tissues (Taylor et al., 2010) indicates significant concurrent up-regulation of 18 (out of a possible 28) gene pairs (*p* values were derived from exact Fisher tests, *p* < 0.05). Red indicates significant mRNA upregulation, and blue indicates significant mRNA downregulation. The bar chart to the right shows that in most patients glycosylation enzyme enzymes tend to be upregulated concurrently with 4–5 other enzymes.

Our meta-analysis made use of two independent clinical RNA validation cohorts, which were analysed using real-time PCR. The first cohort compared RNA samples of benign prostatic hyperplasia (BPH) versus prostate carcinoma obtained from 49 patients (clinical dataset B) (Fig. 3A), and the second cohort compared carcinoma versus adjacent normal tissue within 9 patients (clinical dataset C) (Fig. 3B). 7 out of the 8 glycosylation enzyme genes examined were significantly up-regulated in at least one of these two cohorts, with 5 genes (*ST6GalNAc1*, *GALNT7*, *UAP1*, *CSGALNACT1* and *EDEM3*) significantly up-regulated in both cohorts (*p* < 0.05) (Supplementary Table 7).

Further analysis of our cohort of patient tissue RNA from carcinoma versus normal tissue (clinical dataset C) also showed that several of the 8 glycosylation genes often changed expression in parallel, with the majority of patients showing a concurrent change in 4 or 5 out of the 8 individual genes (Fig. 3B). Analysis of a larger dataset (Taylor et al., 2010) for concurrent expression of the 8 glycosylation enzymes found 18 significant associations (Fig. 3C), suggesting that changes in glycosylation gene expression in PCa generally co-occur for several genes in parallel within tumours.

### 3.3 Androgens Control Expression of *O*-Glycosylation and Core Synthesis Enzymes that are Essential for Viability of PCa Cells

**Fig. 4:**
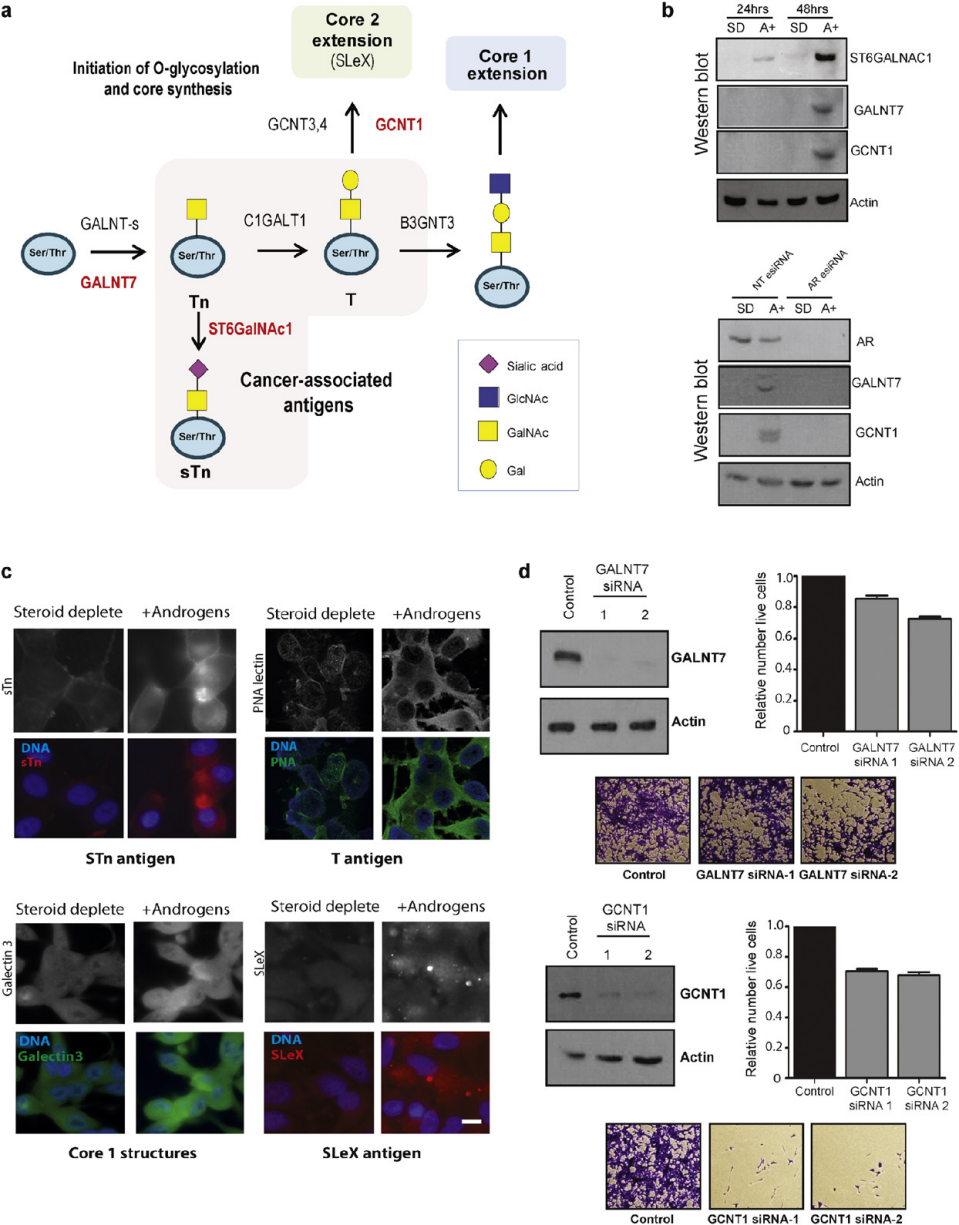
Androgens control expression of *O*-glycosylation and core synthesis enzymes, controlling synthesis of the sTn and SLe^X^ antigens. (A) Overview of the catalytic roles GALNT7, ST6GalNAc1 and GCNT1 play in the initiation of *O*-glycosylation and core synthesis. (B) Western blot analysis of ST6GalNAc1, GALNT7 and GCNT1 enzymes in LNCaP cells grown with 10 nM R1881 (androgens, A +) or without (steroid deplete, SD) for 24 or 48 h. Androgen-regulation of each glycosylation enzyme was confirmed using esiRNA mediated depletion of the AR. Actin was used as a loading control. The specificity of each antibody used was confirmed by detection of siRNA mediated protein depletion (Supplementary Fig. 1). (C) Detection of cancer-associated *O*-glycans in PCa cells, using glycosylation specific antibodies and fluorescently labelled lectins with binding specificities to selected sugar moieties. LNCaP cells grown with 10 nM R1881 (androgens) or without (steroid deplete) for 72 h were labelled with antibodies specific to the glycans shown. A comparison of the images shows that androgens induce dramatic differences in the glycan composition of PCa cells. Consistent with previous data (Munkley et al., 2015c), exposure to androgens upregulates the expression of the cancer-associated sialyl-Tn antigen (sTn). Androgen treatment also upregulates expression of core 1 structures (detected by PNA lectin and Galectin-3 binding) and expression of the Sialyl Lewis X antigen (SLe^X^). Bar is 10 μM. Similar changes in glycan signatures in response to androgen stimulation were also observed in androgen-responsive VCaP prostate cancer cells (Supplementary Fig. 4). (D) GALNT7 and GCNT1 expression is needed for PCa cell viability. For each enzyme siRNA-mediated protein depletion was carried out using two different siRNAs in LNCaP cells grown in full media, and confirmed by western blot after 72 h. 96 h after transfection the relative number of live cells was calculated. Representative crystal violet stained images are shown below. Further details are given in Supplementary Fig. 5.

The above data indicates that androgens control the gene expression of at the transcriptional level of enzymes operating at multiple steps within the glycosylation synthetic pathways. We next tested whether this set of 8 individual glycosylation enzymes are upregulated by androgens at the protein level, and whether these enzymes are functionally important in prostate cancer cells. Three glycosylation enzyme genes, *GALNT7*, *ST6GalNAC1* and *GCNT1* map to the *O*-glycosylation pathway (Fig. 4A). Initiation of *O*-glycosylation is carried out by a family of GALNT sialyltransferase enzymes, including GALNT7, which catalyse the transfer of GalNAc to serine and threonine residues on target proteins to produce the Tn antigen (Ten Hagen et al., 2003). The Tn antigen can be modified by ST6GalNAc1 to produce the truncated cancer-associated sTn antigen (Sewell et al., 2006), or can be converted to core 1 O-glycan structures. Core 1 or the T antigen is then converted to core 2 by one of three core 2 enzymes (GCNT1, 2 and 3). We previously showed that ST6GalNAc1 and the sialyl-Tn (sTn) cancer-associated antigen it produces are directly regulated by androgens in PCa cells (Munkley et al., 2015c, Munkley and Elliott, 2016b). Here, in addition to androgen regulation of ST6GalNAc1,we additionally show androgen control of the O-glycosylation enzymes GALNT7 and GCNT1 (4B, upper panel). Furthermore and consistent with these enzymes being under control of the androgen receptor, protein induction in response to androgen treatment was blocked by siRNA mediated depletion of the AR (Fig. 4B, lower panel). GCNT1 is important for formation of core 2 branched O-glycans and has been implicated in synthesis of the cancer-associated Sialyl Lewis X (SLe^X^) antigen (Chen et al., 2014). As well as upregulation of GCNT1, we also observed increased expression of SLe^X^ in PCa cells in response to androgen stimulation (Fig. 4C, Supplementary Fig. 4), indicating that androgen control of GCNT1 is likely to be an important regulatory step in this glycan synthetic pathway. We also found that in both LNCaP and VCaP cells that androgen exposure leads to up-regulated expression of the core 1 Thomsen-Friedenreich antigen (T-antigen, detected by PNA lectin), which is produced by the GALNT7 enzyme. Also consistent with an up-regulation of core 1 glycans in PCa cells exposed to androgens, we observed an increased binding of the soluble lectin Galectin-3, which binds core 1 glycans (Newlaczyl and Yu, 2011)(Fig. 4C, Supplementary Fig. 4).

To test whether changes in the expression of GALNT7 and GCNT1 contribute to PCa viability, we individually depleted cells of these enzymes using siRNAs. Loss of both enzymes resulted in a significant reduction in cell number and significantly reduced cell viability 96 h after siRNA treatment, in comparison to cells treated with the control siRNA (*p* < 0.02, Fig. 4D and Supplementary Figs. 5 & 6). Taken together, our data suggests that the *O*-glycosylation and core synthesis enzymes GALNT7, ST6GalNAC1 and GCNT1 are induced by androgens in PCa cells at the protein level, and that production of these enzymes could be important for the synthesis of important cancer-associated *O*-glycans and contribute to PCa cell viability.

### 3.4 Essential Enzymes Within the O-GlcNAcylation Pathway are Controlled by Androgens in PCa Cells

**Fig. 5:**
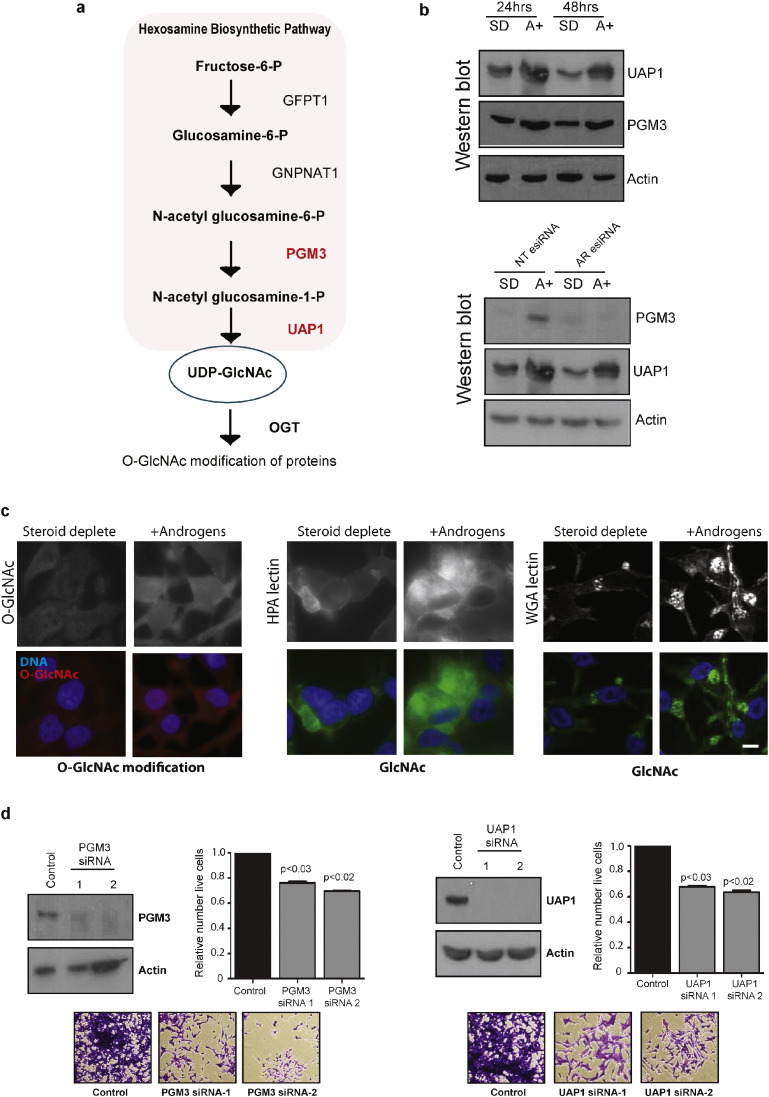
Essential enzymes within the *O*-GlcNAcylation pathway are controlled by androgens in PCa cells. (A) The hexosamine biosynthetic pathway (HBP) catalyses the formation of UDP-GlcNAc and culminates in the attachment of *O*-GlcNAc onto serine/threonine residues of target proteins. HBP enzymes regulated by androgens in prostate cancer cells are shown in red. (B) Western blot analysis of UAP1 and PGM3 enzymes in LNCaP cells grown with 10 nM R1881 (androgens) or without (steroid deplete) for 24 or 48 h. Androgen-regulation of each glycosylation enzyme was confirmed using esiRNA mediated depletion of the AR. Actin expression was monitored as the loading control. The specificity of each antibody used was confirmed by detection of siRNA mediated protein depletion (Supplementary Fig. 1). (C) Detection of *O*-GlcNAcylation in PCa cells, using a glycosylation-specific antibody and fluorescently labelled lectins with binding specificities to O-GlcNAc. LNCaP cells were grown with 10 nM R1881 (androgens) or without (steroid deplete) for 72 h prior to fixation and labelling. A comparison of the images shows that androgens induce an increase in O-GlcNAc in prostate cancer cells. The size bar represents 10 μM. Similar changes in O-GlcNAc expression were also seen in androgen-responsive VCaP prostate cancer cells (Supplementary Fig. 4). (D) UAP1 and PGM3 influence PCa cell growth and viability in vitro. For each enzyme siRNA-mediated protein depletion was carried out using two different siRNAs in LNCaP cells grown in full media, and confirmed by western blot after 72 h. The relative number of live cells at 96 h after transfection was calculated relative to a control non-targeting siRNA. Representative crystal violet stained images are shown below. Further details are given in Supplementary Fig. 5.

The identity of the core set of 8 androgen regulated glycosylation enzymes identified above also suggest that the hexosamine biosynthesis pathway (HBP) is under more extensive androgen control in PCa cells than previously realised. The HBP produces an amino-sugar conjugate UDP-*N*-acetylglucoamine (UDP-GlcNAc), which provides a substrate for posttranslational modification of proteins (Wellen et al., 2010) (Fig. 5A). While it was previously known that the HBP enzyme UAP1 is directly regulated by androgens in PCa cells (Itkonen et al., 2014), our reciprocal analysis demonstrated that the PGM3 enzyme is also androgen-regulated via the AR (Fig. 2, Fig. 5B).

Further supporting the hypothesis that the glycans produced by the HBP are also under androgen control, we detected increased *O*-GlcNAc in PCa cells exposed to androgens (detected by a glycosylation specific antibody and two glycan specific lectins, Fig. 5C and Supplementary Fig. 4). Depletion of either UAP1 or PGM3 with two independent siRNAs significantly reduced cell viability of both LNCaP and CWR22Rv1 cells in comparison to cells treated with the control siRNA, consistent with expression of both these enzymes being important for prostate cancer cell survival (Fig. 5D, Supplementary Figs. 5 & 6).

### 3.5 Androgens Control Synthesis of Chondroitin Sulphate in PCa Cells

**Fig. 6:**
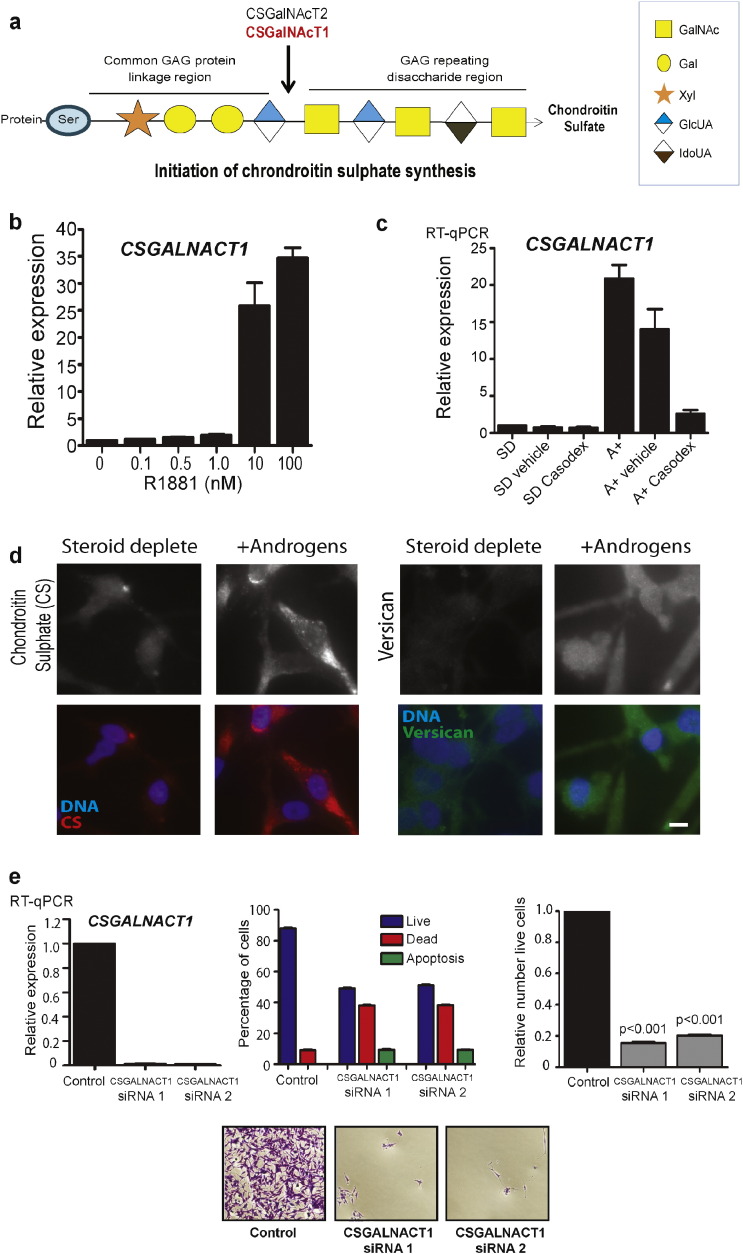
Androgens control synthesis of chondroitin sulphate in PCa cells. (A) Initiation of chondroitin sulphate (CS) synthesis is mediated by two enzymes, CSGalNAcT1 and CSGalNAcT2, of which CSGalNAcT1 is androgen regulated (shown in red). (B) Real-time PCR analysis of *CSGalNAcT1* mRNA over a range of concentrations of R1881 (androgens) in LNCaP cells. (C) Real-time PCR analysis of *CSGalNAcT1* mRNA in LNCaP cells treated with the AR antagonist Casodex^R^ in the presence of 10 nM R1881. (D) Detection of both CS and the CS proteoglycan versican in PCa cells grown with or without androgens (10 nM R1881) for 72 h. Androgen-regulation of the enzyme is for CSGalNAcT1 confirmed at the mRNA level only (we were unable to obtain an antibody against the human CSGalNAcT1 protein which worked in our hands). The size bar is equivalent to 10 μM. Similar changes were also seen in VCaP prostate cancer cells (Supplementary Fig. 4). (E) siRNA-mediated protein depletion of *CSGalNAcT1* in LNCaP cells grown in full media using two different siRNAs was confirmed by real-time PCR after 72 h (upper left panel). The relative number of live, dead and apoptotic cells 96 h after transfection was calculated for each siRNA relative to a control non-targeting siRNA using flow cytometry. Representative crystal violet stained images are shown below.

The identity of the core set of 8 androgen regulated genes we identified by our reciprocal analysis further predicted that synthesis of chondroitin sulphate (CS) is under androgen control in PCa cells, via activation of *CSGalNacT1* expression. While six glycosyltransferases are involved in CS synthesis, the enzyme chondroitin sulphate *N*-acetylgalactosaminyltransferase 1 (CSGalNAcT1) is involved in the initiation and elongation processes (Sakai et al., 2007, Watanabe et al., 2010) (Fig. 6A). We confirmed androgen regulation of *CSGalNacT1* expression in LNCaP and VCaP cells (Fig. 6B, C and Supplementary Fig. 4). In PCa cells treated with androgens we found increased CS synthesis, indicating that *CSGalNacT1* is likely to be a key control point for synthesis of this CS glycan (Fig. 6D left panels). CS forms the GAG side chains of several proteoglycan families, including the PCa associated large CS proteoglycan, Versican. Consistent with previous reports, we also found that the Versican is regulated by androgens in PCa cells (Read et al., 2007) (Fig. 6D right panel). Depletion of *CSGalNAcT1* using two different siRNAs very dramatically reduced CSGALNACT1 mRNA expression as monitored by qPCR. Decreased expression of CSGALNACT1 also increased the numbers of dead and apoptotic cells, and significantly decreased cell viability in both LNCaP and CWR22Rv1 cells in comparison to cells treated with the control siRNA, suggesting a key role for the CSGALNACT1 enzyme in PCa cell biology (Fig. 6E and Supplementary Figs. 5 & 6).

### 3.6 *N*-Glycan Modifications are Controlled by Androgens in Prostate Cancer Cells

**Fig. 7:**
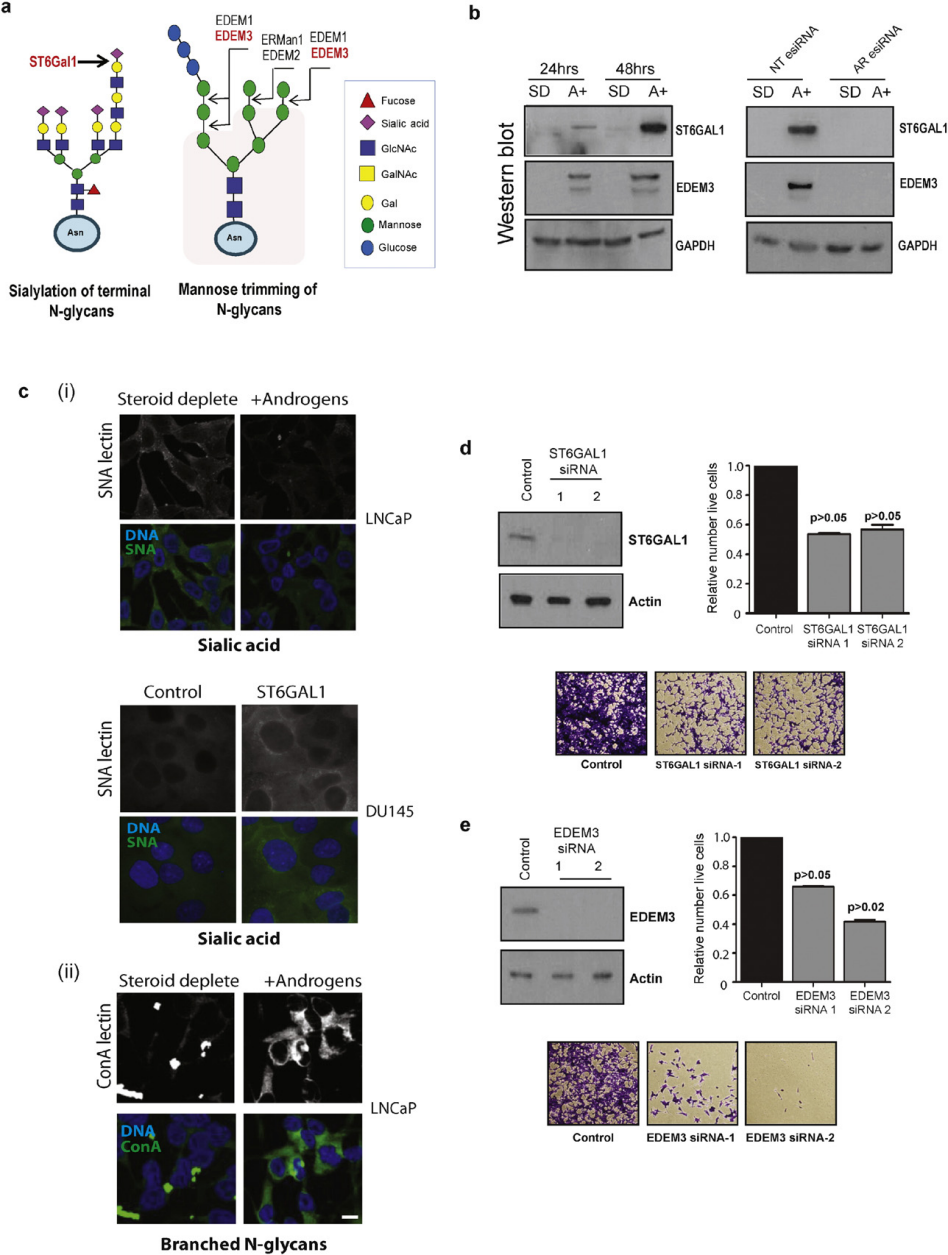
*N*-Glycan modifications are controlled by androgens in prostate cancer cells. (A) ST6GAL1 is a sialytransferase which catalyses the addition of terminal sialic acid residues onto *N*-glycans. EDEM3 mediates the trimming of N-glycans, which are then further modified by other enzymes. (B) Western blot analysis of ST6GAL1 and EDEM3 in LNCaP cells grown with 10 nM R1881 (androgens) or without (steroid deplete) for 24 or 48 h. Androgen-regulation of ST6GAL1 and EDEM3 was confirmed using esiRNA mediated depletion of the AR, which blocks up-regulation of both proteins. GAPDH was used as a loading control. (C) (i) Detection of sialic acid in LNCaP cells grown with or without androgens for 72 h, and in (ii) DU145 cells over-expressing either EV control or ST6GAL1 using SNA lectin. Although overall sialic acid expression did not increase with exposure to androgens in LNCaP cells, over-expression of ST6GAL1 in DU145 cells does result in an increase. (iii) Detection of branched N-glycans in LNCaP cells using fluorescently labelled ConA lectin. Cells treated with androgens (10 nM R1881) for 72 h have increased ConA binding relative to cells grown without androgens (steroid deplete). (D,E) siRNA-mediated protein depletion of ST6GAL1 and EDEM3 in LNCaP cells grown in full media using two different siRNAs was confirmed by western blot after 72 h. The relative number of live, dead and apoptotic cells 96 h after transfection was relative to a control non-targeting siRNA using flow cytometry (upper right panels). Representative crystal violet stained images are shown below.

Our data further predicted that that N-glycan synthesis is also under androgen control in PCa cells, via expression control of the *ST6GAL1* and *EDEM3* genes. *ST6GAL1* encodes a sialyltransferase that catalyses the transfer of sialic acid onto terminal galactose containing N-glycan substrates (Schultz et al., 2013, Hedlund et al., 2008) (Fig. 7A). We confirmed expression of ST6GAL1 protein is regulated by androgens in PCa cells by western blot (Fig. 7B). While we observed an increase in ST6GAL1 expression in both LNCaP and VCaP cells in response to androgen stimulation, there was no corresponding increase in sialylation of terminal N-glycans detected by SNA binding (Fig. 7Ci and Supplementary Fig. 4) suggesting the influence of this enzyme on terminal sialylation may be dependent upon cellular background. Supporting such a model, expression of ST6GAL1 in DU145 PCa cells did increase SNA binding (Fig. 7Cii). Although it did not detectably increase sialylation of terminal N-glycans, depletion of ST6GAL1 using two different siRNAs led to reduced cell viability in both LNCaP and CWR22Rv1 cells (Fig. 7D and Supplementary Figs. 5 & 6) indicating ST6GAL1 has an important biological role in these cell lines.

EDEM3 stimulates mannose trimming of N-glycans from total glycoproteins, and also enhances glycoprotein endoplasmic reticulum-associated degradation (ERAD) of misfolded glycoproteins (Hirao et al., 2006, Olivari and Molinari, 2007) (Fig. 7A), which is particularly important in the context of cancer where increased metabolic needs lead to the accumulation of faulty proteins (Olzmann et al., 2013). We confirmed androgen regulation of EDEM3 protein expression in PCa cells (Fig. 7B). Consistent with this we also found that an increase in branched N-glycans (detected by ConA lectin) in response to androgen treatment (Fig. 7Ciii). siRNA depletion experiments with two independent siRNAs showed that expression of EDEM3 enzyme is important for PCa cell viability and growth (Fig. 7E and Supplementary Figs. 5 & 6).

## 4 Discussion

Although aberrant glycosylation is common in cancer and has been linked to PCa progression (Munkley et al., 2016), our understanding of what drives these cancer associated glycan changes has been scant and has relied upon anecdotal observations. Here, using a global transcriptomic analysis to identify genes that have a reciprocal expression signature between androgen deprivation therapy in patients and androgen stimulation in culture, we identify glycosylation as a cohesive enriched pathway that is under extensive control by androgen hormones. A total of 31 genes encoding glycosylation enzymes were revealed through reciprocal expression signatures, and of these we further identify a core set of 8 androgen regulated glycosylation enzymes that are also significantly up-regulated in multiple cohorts of clinical PCa material. Significantly, only two of these 8 genes, *ST6GalNAc1* and *UAP1*, have previously been identified to have roles in clinical PCa (Munkley et al., 2015c, Itkonen et al., 2014). The remaining 6 genes are identified as androgen regulated by our dataset, and reveal new control points through which these glycan synthesis pathways can be regulated in cells, and new mechanisms through which androgens regulate the behaviour of PCa cells. We find each of these 8 glycosylation enzymes are important for PCa cell viability, suggesting that loss of these enzymes in response to ADT might make an important and previously unknown contribution to tumour regression in patients.

Glycosylated proteins and other glycoconjugates are major cellular components which have been causally associated with all of the hallmarks of cancer (Munkley and Elliott, 2016a). Altered activity or expression of glycosylation enzymes in cancer cells can lead to glycan modifications to alter cell-cell adhesion, migration, interactions with the cell matrix, immune surveillance, signalling and cellular metabolism and may modify these processes in PCa (Munkley et al., 2016). Glycosylation pathways produce glycan structures via the cumulative enzymatic activity of many glycosylation enzymes. The core set of 8 androgen regulated glycosylation genes that we identify here belong to the *O*-glycan, HBP, chondroitin sulphate, and *N*-glycan synthetic pathways that are already known to be important in cancer.

*O*-glycans are altered in the early stages of cellular transformation, and are important for cancer initiation, invasion and metastasis (Pinho and Reis, 2015). We showed previously that induction of the *O*-glycosylation enzyme ST6GalNAc1 by androgens in prostate cancer cells can reduce prostate cancer cell adhesion (Munkley et al., 2015c, Munkley and Elliott, 2016b). In this study we confirm androgen regulation of ST6GalNAc1 and show for the first time a link between androgens and two additional O-glycosylation enzymes in prostate cancer, GALNT7 and GCNT1. Expression of GALNT7 has previously been found to be up-regulated in several cancer types, including PCa (Chen et al., 2014, Li et al., 2014, Peng et al., 2012, Gaziel-Sovran et al., 2011), and is linked to metastasis and invasion in nasopharyngeal carcinoma (Nie et al., 2015). As GALNT7 initiates O-glycosylation to produce the Tn antigen, upregulation of this enzyme could potentially be linked to a range of changes in O-glycans in prostate cancer cells. The Tn antigen can be further modified by ST6GalNAc1 to produce sTn raising the possibility for a role for GALNT7 in the expression of this important cancer associated antigen. GALNT7 may also have a role in the synthesis of the T-antigen, which is frequently over-expressed in cancer (Hanisch and Baldus, 1997, Cazet et al., 2010, Spinger, 1984) and is associated with the adhesion of PCa cells to the endothelium (Glinsky et al., 2001). In cancer, truncated *O*-glycans often recruit carbohydrate binding proteins, or lectins that can play a key role in disease progression and metastasis (Munkley, 2016). Here we show increased binding of Galectin-3 in prostate cancer cells treated with androgens. These results are important, as Galectin-3 has been implicated in PCa tumour growth, and suggested as a potential therapeutic target (Wang et al., 2009b). Our data are also the first to suggest a link between the AR and expression of GCNT1 in prostate cancer. Increased GCNT1 expression has already been linked to PCa progression, shown to increase prostate tumour growth in vivo, and been implicated in synthesis of the cancer-associated SLe^X^ antigen (Chen et al., 2014, Hagisawa et al., 2005, Kojima et al., 2015). Here, we show for the first time that expression of both GCNT1 and the SLe^X^ antigen are regulated by androgens in prostate cancer cells. SLe^X^ is a major sialylated antigen associated with poor prognosis and metastasis in PCa, and has been detected on PSA and MUC1 proteins from patients with PCa (Chen et al., 2014).

Multiple levels of evidence presented here also suggest that in addition to the *O*-glycan pathway, three other major glycosylation pathways important in PCa are under the control of androgens. Firstly, one of these pathways is the HBP, which creates the amino-sugar conjugate *O*-GlcNAc which is known to be elevated in PCa (Kamigaito et al., 2014). O-GlcNAc is so important it has been suggested as a new hallmark of cancer (Fardini et al., 2013). Our data shows that expression levels of the two final two enzymes in the HBP are controlled by androgens, as is the expression of the O-GlcNAc glycan itself. Secondly, we also detect androgen regulation of the pathway producing chondroitin sulphate (CS) and of CS itself, which is a type of GAG (glycosaminoglycan) present in extracellular matrices and on the surface of many cell types (Gandhi and Mancera, 2008). CS is increased in metastatic PCa (Ricciardelli et al., 2009a, Ricciardelli et al., 1997), where it is thought to play a role in cellular proliferation and differentiation (Hardingham and Fosang, 1992). Expression of the large CS proteoglycan Versican, which is linked to cell adhesion and associated with poor outcomes in many different cancers including PCa (Ricciardelli et al., 2009b), also responds to androgens.

Thirdly, we also find that androgens control production of two key enzymes involved in *N*-glycan processing; ST6GAL1 and EDEM3. ST6GAl1 is important for controlling sialylation of *N*-terminal glycans, and is over-expressed in many types of cancer (Swindall et al., 2013). Altered sialylation has long been associated with cancer cell metastasis, invasion and survival (Pinho and Reis, 2015, Munkley, 2016). Expression of ST6GAL1 is already linked to the epithelial-mesenchymal transition (EMT) and malignancy (Lu et al., 2014), but the mechanisms driving these changes remain poorly understood. EDEM3 controls mannose trimming of N-glycans, and has previously been shown to be upregulated in malignant PCa as part of a glycosylation gene signature (Chen et al., 2014). β1,6GlcNAc tri/tetra branched N-glycans and cryptic N-glycans are detected in PCa and have been linked to metastasis and are being investigated as potential biomarkers (Ishibashi et al., 2014, Wang et al., 2013). It is possible that the elevated levels of EDEM3 plays a role in creating these aberrant N-glycans.

PCa is a unique and confounding disease, characterised by prognostic heterogeneity and there is a key clinical need to develop biomarkers to help distinguish indolent from aggressive disease, as well as to develop new treatments for advanced PCa. Our study identifying glycosylation as an androgen-regulated process in PCa has important clinical implications both diagnostically and therapeutically. The most widely used serological biomarkers for cancer diagnosis and monitoring are glycoproteins, and monitoring the glycan composition of specific glycoproteins can dramatically increase their specificity as biomarkers (Reis et al., 2010). Numerous studies have examined whether a tumour specific glycan signature on prostate specific antigen (PSA) can be used to distinguish between BPH and PCa (Gilgunn et al., 2013) and there are ongoing studies analysing the glycan composition of serum, plasma, exosomes, expressed-prostatic secretions (EPS) fluids and formalin-fixed, paraffin-embedded (FFPE) tissues to diagnose PCa and determine prognosis (Yoneyama et al., 2014, Kyselova et al., 2007, Drake and Kislinger, 2014, Powers et al., 2013, Powers et al., 2014, Drake et al., 2015). Our study identifies a panel of glycosylation enzymes and their corresponding antigens as potentially important in prostate cancer. An increased understanding of how glycosylation influences prostate cancer cell behaviour should aid in the development of glycosylation specific biomarkers for use in the early detection and management of PCa. Glycans also likely play roles in all aspects of cancer progression and are therefore attractive targets for therapeutic intervention (Munkley and Elliott, 2016a). Previous work has shown that inhibiting glycosylation, even in conditions where the AR is active, can reduce PCa cell viability (Itkonen and Mills, 2013). An increased understanding of how glycosylation modulates the biological function of prostate cancer cells will allow the development of a relatively unexploited field of drugs based on inhibitors, glycan antagonists and glycan function modulators.

## Appendix A Supplementary data

Supplementary figures. Image 1

Supplementary tables. Image 2

## References

[bb0005] Barfeld S.J., Itkonen H.M., Urbanucci A., Mills I.G. (2014). Androgen-regulated metabolism and biosynthesis in prostate cancer. Endocr. Relat. Cancer.

[bb0010] Cai C., He H.H., Chen S., Coleman I., Wang H., Fang Z., Chen S., Nelson P.S., Liu X.S., Brown M., Balk S.P. (2011). Androgen receptor gene expression in prostate cancer is directly suppressed by the androgen receptor through recruitment of lysine-specific demethylase 1. Cancer Cell.

[bb2000] Cazet (2010). Breast Cancer Res..

[bb0015] Chen Z., Gulzar Z.G., St Hill C.A., Walcheck B., Brooks J.D. (2014). Increased expression of GCNT1 is associated with altered *O*-glycosylation of PSA, PAP, and MUC1 in human prostate cancers. Prostate.

[bb0020] Craft N., Shostak Y., Carey M., Sawyers C.L. (1999). A mechanism for hormone-independent prostate cancer through modulation of androgen receptor signaling by the HER-2/neu tyrosine kinase. Nat. Med..

[bb0025] Drake R.R., Kislinger T. (2014). The proteomics of prostate cancer exosomes. Expert Rev. Proteomics.

[bb0030] Drake R.R., Jones E.E., Powers T.W., Nyalwidhe J.O. (2015). Chapter ten – altered glycosylation in prostate cancer. Adv. Cancer Res..

[bb0035] Fardini Y., Dehennaut V., Lefebvre T., Issad T. (2013). *O*-GlcNAcylation: a new cancer hallmark?. Front. Endocrinol..

[bb0040] Gandhi N.S., Mancera R.L. (2008). The structure of glycosaminoglycans and their interactions with proteins. Chem. Biol. Drug Des..

[bb0045] Gaziel-Sovran A., Segura M.F., Di Micco R., Collins M.K., Hanniford D., Vega-Saenz De Miera E., Rakus J.F., Dankert J.F., Shang S., Kerbel R.S., Bhardwaj N., Shao Y., Darvishian F., Zavadil J., Erlebacher A., Mahal L.K., Osman I., Hernando E. (2011). miR-30b/30d regulation of GalNAc transferases enhances invasion and immunosuppression during metastasis. Cancer Cell.

[bb0050] Gilgunn S., Conroy P.J., Saldova R., Rudd P.M., O'kennedy R.J. (2013). Aberrant PSA glycosylation—a sweet predictor of prostate cancer. Nat. Rev. Urol..

[bb0055] Glinsky V.V., Glinsky G.V., Rittenhouse-Olson K., Huflejt M.E., Glinskii O.V., Deutscher S.L., Quinn T.P. (2001). The role of Thomsen-Friedenreich antigen in adhesion of human breast and prostate cancer cells to the endothelium. Cancer Res..

[bb0060] Grasso C.S., Wu Y.M., Robinson D.R., Cao X., Dhanasekaran S.M., Khan A.P., Quist M.J., Jing X., Lonigro R.J., Brenner J.C., Asangani I.A., Ateeq B., Chun S.Y., Siddiqui J., Sam L., Anstett M., Mehra R., Prensner J.R., Palanisamy N., Ryslik G.A., Vandin F., Raphael B.J., Kunju L.P., Rhodes D.R., Pienta K.J., Chinnaiyan A.M., Tomlins S.A. (2012). The mutational landscape of lethal castration-resistant prostate cancer. Nature.

[bb0065] Haag P., Bektic J., Bartsch G., Klocker H., Eder I.E. (2005). Androgen receptor down regulation by small interference RNA induces cell growth inhibition in androgen sensitive as well as in androgen independent prostate cancer cells. J. Steroid Biochem. Mol. Biol..

[bb0070] Hagisawa S., Ohyama C., Takahashi T., Endoh M., Moriya T., Nakayama J., Arai Y., Fukuda M. (2005). Expression of core 2 beta1,6-*N*-acetylglucosaminyltransferase facilitates prostate cancer progression. Glycobiology.

[bb2200] Hanisch F.G., Baldus S.E. (1997). The Thomsen-Freidenreich (TF) antigen. Histol. Histopathol..

[bb0075] Hara T., Miyazaki H., Lee A., Tran C.P., Reiter R.E. (2008). Androgen receptor and invasion in prostate cancer. Cancer Res..

[bb0080] Hardingham T.E., Fosang A.J. (1992). Proteoglycans: many forms and many functions. FASEB J..

[bb0085] Hedlund M., Ng E., Varki A., Varki N.M. (2008). Alpha 2-6-linked sialic acids on *N*-glycans modulate carcinoma differentiation in vivo. Cancer Res..

[bb0090] Hirao K., Natsuka Y., Tamura T., Wada I., Morito D., Natsuka S., Romero P., Sleno B., Tremblay L.O., Herscovics A., Nagata K., Hosokawa N. (2006). EDEM3, a soluble EDEM homolog, enhances glycoprotein endoplasmic reticulum-associated degradation and mannose trimming. J. Biol. Chem..

[bb0095] Ishibashi Y., Tobisawa Y., Hatakeyama S., Ohashi T., Tanaka M., Narita S., Koie T., Habuchi T., Nishimura S., Ohyama C., Yoneyama T. (2014). Serum tri- and tetra-antennary *N*-glycan is a potential predictive biomarker for castration-resistant prostate cancer. Prostate.

[bb0100] Itkonen H.M., Mills I.G. (2013). *N*-linked glycosylation supports cross-talk between receptor tyrosine kinases and androgen receptor. PLoS One.

[bb0105] Itkonen H.M., Engedal N., Babaie E., Luhr M., Guldvik I.J., Minner S., Hohloch J., Tsourlakis M.C., Schlomm T., Mills I.G. (2014). UAP1 is overexpressed in prostate cancer and is protective against inhibitors of *N*-linked glycosylation. Oncogene.

[bb0110] Kamigaito T., Okaneya T., Kawakubo M., Shimojo H., Nishizawa O., Nakayama J. (2014). Overexpression of *O*-GlcNAc by prostate cancer cells is significantly associated with poor prognosis of patients. Prostate Cancer Prostatic Dis..

[bb0115] Karantanos T., Corn P.G., Thompson T.C. (2013). Prostate cancer progression after androgen deprivation therapy: mechanisms of castrate resistance and novel therapeutic approaches. Oncogene.

[bb0120] Kim D., Pertea G., Trapnell C., Pimentel H., Kelley R., Salzberg S.L. (2013). TopHat2: accurate alignment of transcriptomes in the presence of insertions, deletions and gene fusions. Genome Biol..

[bb0125] Kojima Y., Yoneyama T., Hatakeyama S., Mikami J., Sato T., Mori K., Hashimoto Y., Koie T., Ohyama C., Fukuda M., Tobisawa Y. (2015). Detection of core2 beta-1.6-*N*-acetylglucosaminyltransferase in post-digital rectal examination urine is a reliable indicator for extracapsular extension of prostate cancer. PLoS One.

[bb0130] Kyselova Z., Mechref Y., Al Bataineh M.M., Dobrolecki L.E., Hickey R.J., Vinson J., Sweeney C.J., Novotny M.V. (2007). Alterations in the serum glycome due to metastatic prostate cancer. J. Proteome Res..

[bb0135] Langmead B., Salzberg S.L. (2012). Fast gapped-read alignment with Bowtie 2. Nat. Methods.

[bb0140] Li W., Ma H., Sun J. (2014). MicroRNA34a/c function as tumor suppressors in Hep2 laryngeal carcinoma cells and may reduce GALNT7 expression. Mol. Med. Rep..

[bb0145] Liu Y. (2006). Fatty acid oxidation is a dominant bioenergetic pathway in prostate cancer. Prostate Cancer Prostatic Dis..

[bb0150] Lu J., Isaji T., Im S., Fukuda T., Hashii N., Takakura D., Kawasaki N., Gu J. (2014). Beta-galactoside alpha2,6-sialyltranferase 1 promotes transforming growth factor-beta-mediated epithelial-mesenchymal transition. J. Biol. Chem..

[bb0155] Massie C.E., Adryan B., Barbosa-Morais N.L., Lynch A.G., Tran M.G., Neal D.E., Mills I.G. (2007). New androgen receptor genomic targets show an interaction with the ETS1 transcription factor. EMBO Rep..

[bb0160] Massie C.E., Lynch A., Ramos-Montoya A., Boren J., Stark R., Fazli L., Warren A., Scott H., Madhu B., Sharma N., Bon H., Zecchini V., Smith D.M., Denicola G.M., Mathews N., Osborne M., Hadfield J., Macarthur S., Adryan B., Lyons S.K., Brindle K.M., Griffiths J., Gleave M.E., Rennie P.S., Neal D.E., Mills I.G. (2011). The androgen receptor fuels prostate cancer by regulating central metabolism and biosynthesis. EMBO J..

[bb0165] Mills I.G. (2014). Maintaining and reprogramming genomic androgen receptor activity in prostate cancer. Nat. Rev. Cancer.

[bb0170] Munkley J. (2016). The role of sialyl-Tn in cancer. Int. J. Mol. Sci..

[bb0180] Munkley J., Elliott D.J. (2016). Hallmarks of glycosylation in cancer. Oncotarget.

[bb0185] Munkley J., Elliott D.J. (2016). Sugars and cell adhesion: the role of ST6GalNAc1 in prostate cancer progression. Cancer Cell Microenviron..

[bb0190] Munkley J., Rajan P., Lafferty N.P., Dalgliesh C., Jackson R.M., Robson C.N., Leung H.Y., Elliott D.J. (2014). A novel androgen-regulated isoform of the TSC2 tumour suppressor gene increases cell proliferation. Oncotarget.

[bb0195] Munkley J., Lafferty N.P., Kalna G., Robson C.N., Leung H.Y., Rajan P., Elliott D.J. (2015). Androgen-regulation of the protein tyrosine phosphatase PTPRR activates ERK1/2 signalling in prostate cancer cells. BMC Cancer.

[bb0200] Munkley J., Livermore K.E., Mcclurg U.L., Kalna G., Knight B., Mccullagh P., Mcgrath J., Crundwell M., Leung H.Y., Robson C.N., Harries L.W., Rajan P., Elliott D.J. (2015). The PI3K regulatory subunit gene PIK3R1 is under direct control of androgens and repressed in prostate cancer cells. Oncoscience.

[bb0205] Munkley J., Oltean S., Vodak D., Wilson B.T., Livermore K.E., Zhou Y., Star E., Floros V.I., Johannessen B., Knight B., Mccullagh P., Mcgrath J., Crundwell M., Skotheim R.I., Robson C.N., Leung H.Y., Harries L.W., Rajan P., Mills I.G., Elliott D.J. (2015). The androgen receptor controls expression of the cancer-associated sTn antigen and cell adhesion through induction of ST6GalNAc1 in prostate cancer. Oncotarget.

[bb0210] Munkley J., Mills I.G., Elliott D.J. (2016). The role of glycans in the development and progression of prostate cancer. Nat. Rev. Urol..

[bb0215] Newlaczyl A.U., Yu L.G. (2011). Galectin-3—a jack-of-all-trades in cancer. Cancer Lett..

[bb0220] Nie G.H., Luo L., Duan H.F., Li X.Q., Yin M.J., Li Z., Zhang W. (2015). GALNT7, a Target of miR-494, Participates in the Oncogenesis of Nasopharyngeal Carcinoma.

[bb0225] Olivari S., Molinari M. (2007). Glycoprotein folding and the role of EDEM1, EDEM2 and EDEM3 in degradation of folding-defective glycoproteins. FEBS Lett..

[bb0230] Olzmann J.A., Kopito R.R., Christianson J.C. (2013). The mammalian endoplasmic reticulum-associated degradation system. Cold Spring Harb. Perspect. Biol..

[bb0235] Peng R.Q., Wan H.Y., Li H.F., Liu M., Li X., Tang H. (2012). MicroRNA-214 suppresses growth and invasiveness of cervical cancer cells by targeting UDP-*N*-acetyl-alpha-*D*-galactosamine:polypeptide *N*-acetylgalactosaminyltransferase 7. J. Biol. Chem..

[bb0240] Pinho S.S., Reis C.A. (2015). Glycosylation in cancer: mechanisms and clinical implications. Nat. Rev. Cancer.

[bb0245] Powers T.W., Jones E.E., Betesh L.R., Romano P.R., Gao P., Copland J.A., Mehta A.S., Drake R.R. (2013). Matrix assisted laser desorption ionization imaging mass spectrometry workflow for spatial profiling analysis of *N*-linked glycan expression in tissues. Anal. Chem..

[bb0250] Powers T.W., Neely B.A., Shao Y., Tang H., Troyer D.A., Mehta A.S., Haab B.B., Drake R.R. (2014). MALDI imaging mass spectrometry profiling of N-glycans in formalin-fixed paraffin embedded clinical tissue blocks and tissue microarrays. PLoS One.

[bb0255] Rajan P., Dalgliesh C., Carling P.J., Buist T., Zhang C., Grellscheid S.N., Armstrong K., Stockley J., Simillion C., Gaughan L., Kalna G., Zhang M.Q., Robson C.N., Leung H.Y., Elliott D.J. (2011). Identification of novel androgen-regulated pathways and mRNA isoforms through genome-wide exon-specific profiling of the LNCaP transcriptome. PLoS One.

[bb0260] Rajan P., Sudbery I.M., Villasevil M.E., Mui E., Fleming J., Davis M., Ahmad I., Edwards J., Sansom O.J., Sims D., Ponting C.P., Heger A., Mcmenemin R.M., Pedley I.D., Leung H.Y. (2014). Next-generation sequencing of advanced prostate cancer treated with androgen-deprivation therapy. Eur. Urol..

[bb0265] Read J.T., Rahmani M., Boroomand S., Allahverdian S., Mcmanus B.M., Rennie P.S. (2007). Androgen receptor regulation of the versican gene through an androgen response element in the proximal promoter. J. Biol. Chem..

[bb0270] Reis C.A., Osorio H., Silva L., Gomes C., David L. (2010). Alterations in glycosylation as biomarkers for cancer detection. J. Clin. Pathol..

[bb0275] Ricciardelli C., Mayne K., Sykes P.J., Raymond W.A., Mccaul K., Marshall V.R., Tilley W.D., Skinner J.M., Horsfall D.J. (1997). Elevated stromal chondroitin sulfate glycosaminoglycan predicts progression in early-stage prostate cancer. Clin. Cancer Res..

[bb0280] Ricciardelli C., Sakko A.J., Stahl J., Tilley W.D., Marshall V.R., Horsfall D.J. (2009). Prostatic chondroitin sulfate is increased in patients with metastatic disease but does not predict survival outcome. Prostate.

[bb0285] Ricciardelli C., Sakko A.J., Ween M.P., Russell D.L., Horsfall D.J. (2009). The biological role and regulation of versican levels in cancer. Cancer Metastasis Rev..

[bb0290] Robinson D., Van Allen E.M., Wu Y.M., Schultz N., Lonigro R.J., Mosquera J.M., Montgomery B., Taplin M.E., Pritchard C.C., Attard G., Beltran H., Abida W., Bradley R.K., Vinson J., Cao X., Vats P., Kunju L.P., Hussain M., Feng F.Y., Tomlins S.A., Cooney K.A., Smith D.C., Brennan C., Siddiqui J., Mehra R., Chen Y., Rathkopf D.E., Morris M.J., Solomon S.B., Durack J.C., Reuter V.E., Gopalan A., Gao J., Loda M., Lis R.T., Bowden M., Balk S.P., Gaviola G., Sougnez C., Gupta M., Yu E.Y., Mostaghel E.A., Cheng H.H., Mulcahy H., True L.D., Plymate S.R., Dvinge H., Ferraldeschi R., Flohr P., Miranda S., Zafeiriou Z., Tunariu N., Mateo J., Perez-Lopez R., Demichelis F., Robinson B.D., Schiffman M., Nanus D.M., Tagawa S.T., Sigaras A., Eng K.W., Elemento O., Sboner A., Heath E.I., Scher H.I., Pienta K.J., Kantoff P., De Bono J.S., Rubin M.A., Nelson P.S., Garraway L.A., Sawyers C.L., Chinnaiyan A.M. (2015). Integrative clinical genomics of advanced prostate cancer. Cell.

[bb0295] Sakai K., Kimata K., Sato T., Gotoh M., Narimatsu H., Shinomiya K., Watanabe H. (2007). Chondroitin sulfate *N*-acetylgalactosaminyltransferase-1 plays a critical role in chondroitin sulfate synthesis in cartilage. J. Biol. Chem..

[bb0300] Schultz M.J., Swindall A.F., Wright J.W., Sztul E.S., Landen C.N., Bellis S.L. (2013). ST6Gal-I sialyltransferase confers cisplatin resistance in ovarian tumor cells. J. Ovarian Res..

[bb0305] Segawa T., Nau M.E., Xu L.L., Chilukuri R.N., Makarem M., Zhang W., Petrovics G., Sesterhenn I.A., Mcleod D.G., Moul J.W., Vahey M., Srivastava S. (2002). Androgen-induced expression of endoplasmic reticulum (ER) stress response genes in prostate cancer cells. Oncogene.

[bb0310] Sewell R., Backstrom M., Dalziel M., Gschmeissner S., Karlsson H., Noll T., Gatgens J., Clausen H., Hansson G.C., Burchell J., Taylor-Papadimitriou J. (2006). The ST6GalNAc-I sialyltransferase localizes throughout the Golgi and is responsible for the synthesis of the tumor-associated sialyl-Tn *O*-glycan in human breast cancer. J. Biol. Chem..

[bb0315] Sharma N.L., Massie C.E., Ramos-Montoya A., Zecchini V., Scott H.E., Lamb A.D., Macarthur S., Stark R., Warren A.Y., Mills I.G., Neal D.E. (2013). The androgen receptor induces a distinct transcriptional program in castration-resistant prostate cancer in man. Cancer Cell.

[bb0320] Sheng X., Arnoldussen Y.J., Storm M., Tesikova M., Nenseth H.Z., Zhao S., Fazli L., Rennie P., Risberg B., Waehre H., Danielsen H., Mills I.G., Jin Y., Hotamisligil G., Saatcioglu F. (2015). Divergent androgen regulation of unfolded protein response pathways drives prostate cancer. EMBO Mol. Med..

[bb0325] Snoek R., Cheng H., Margiotti K., Wafa L.A., Wong C.A., Wong E.C., Fazli L., Nelson C.C., Gleave M.E., Rennie P.S. (2009). In vivo knockdown of the androgen receptor results in growth inhibition and regression of well-established, castration-resistant prostate tumors. Clin. Cancer Res..

[bb2100] Spinger G.F. (1984). T and Tn, general carcinoma antigens. Science.

[bb0330] Steinkamp M.P., O'mahony O.A., Brogley M., Rehman H., Lapensee E.W., Dhanasekaran S., Hofer M.D., Kuefer R., Chinnaiyan A., Rubin M.A., Pienta K.J., Robins D.M. (2009). Treatment-dependent androgen receptor mutations in prostate cancer exploit multiple mechanisms to evade therapy. Cancer Res..

[bb0335] Suburu J., Chen Y.Q. (2012). Lipids and prostate cancer. Prostaglandins Other Lipid Mediat..

[bb0340] Sun S., Sprenger C.C., Vessella R.L., Haugk K., Soriano K., Mostaghel E.A., Page S.T., Coleman I.M., Nguyen H.M., Sun H., Nelson P.S., Plymate S.R. (2010). Castration resistance in human prostate cancer is conferred by a frequently occurring androgen receptor splice variant. J. Clin. Invest..

[bb0345] Swindall A.F., Londono-Joshi A.I., Schultz M.J., Fineberg N., Buchsbaum D.J., Bellis S.L. (2013). ST6Gal-I protein expression is upregulated in human epithelial tumors and correlates with stem cell markers in normal tissues and colon cancer cell lines. Cancer Res..

[bb0350] Swinnen J.V., Esquenet M., Goossens K., Heyns W., Verhoeven G. (1997). Androgens stimulate fatty acid synthase in the human prostate cancer cell line LNCaP. Cancer Res..

[bb0355] Swinnen J.V., Ulrix W., Heyns W., Verhoeven G. (1997). Coordinate regulation of lipogenic gene expression by androgens: evidence for a cascade mechanism involving sterol regulatory element binding proteins. Proc. Natl. Acad. Sci. U. S. A..

[bb0360] Swinnen J.V., Vanderhoydonc F., Elgamal A.A., Eelen M., Vercaeren I., Joniau S., Van Poppel H., Baert L., Goossens K., Heyns W., Verhoeven G. (2000). Selective activation of the fatty acid synthesis pathway in human prostate cancer. Int. J. Cancer.

[bb0365] Taylor B.S., Schultz N., Hieronymus H., Gopalan A., Xiao Y., Carver B.S., Arora V.K., Kaushik P., Cerami E., Reva B., Antipin Y., Mitsiades N., Landers T., Dolgalev I., Major J.E., Wilson M., Socci N.D., Lash A.E., Heguy A., Eastham J.A., Scher H.I., Reuter V.E., Scardino P.T., Sander C., Sawyers C.L., Gerald W.L. (2010). Integrative genomic profiling of human prostate cancer. Cancer Cell.

[bb0370] Ten Hagen K.G., Fritz T.A., Tabak L.A. (2003). All in the family: the UDP-GalNAc:polypeptide *N*-acetylgalactosaminyltransferases. Glycobiology.

[bb0375] Trapnell C., Roberts A., Goff L., Pertea G., Kim D., Kelley D.R., Pimentel H., Salzberg S.L., Rinn J.L., Pachter L. (2012). Differential gene and transcript expression analysis of RNA-seq experiments with TopHat and Cufflinks. Nat. Protoc..

[bb0380] Trapnell C., Hendrickson D.G., Sauvageau M., Goff L., Rinn J.L., Pachter L. (2013). Differential analysis of gene regulation at transcript resolution with RNA-seq. Nat. Biotechnol..

[bb0385] Varambally S., Yu J., Laxman B., Rhodes D.R., Mehra R., Tomlins S.A., Shah R.B., Chandran U., Monzon F.A., Becich M.J., Wei J.T., Pienta K.J., Ghosh D., Rubin M.A., Chinnaiyan A.M. (2005). Integrative genomic and proteomic analysis of prostate cancer reveals signatures of metastatic progression. Cancer Cell.

[bb0390] Veldscholte J., Ris-Stalpers C., Kuiper G.G., Jenster G., Berrevoets C., Claassen E., Van Rooij H.C., Trapman J., Brinkmann A.O., Mulder E. (1990). A mutation in the ligand binding domain of the androgen receptor of human LNCaP cells affects steroid binding characteristics and response to anti-androgens. Biochem. Biophys. Res. Commun..

[bb0395] Visakorpi T., Hyytinen E., Koivisto P., Tanner M., Keinanen R., Palmberg C., Palotie A., Tammela T., Isola J., Kallioniemi O.P. (1995). In vivo amplification of the androgen receptor gene and progression of human prostate cancer. Nat. Genet..

[bb0400] Wang Q., Li W., Liu X.S., Carroll J.S., Janne O.A., Keeton E.K., Chinnaiyan A.M., Pienta K.J., Brown M. (2007). A hierarchical network of transcription factors governs androgen receptor-dependent prostate cancer growth. Mol. Cell.

[bb0405] Wang Q., Li W., Zhang Y., Yuan X., Xu K., Yu J., Chen Z., Beroukhim R., Wang H., Lupien M., Wu T., Regan M.M., Meyer C.A., Carroll J.S., Manrai A.K., Janne O.A., Balk S.P., Mehra R., Han B., Chinnaiyan A.M., Rubin M.A., True L., Fiorentino M., Fiore C., Loda M., Kantoff P.W., Liu X.S., Brown M. (2009). Androgen receptor regulates a distinct transcription program in androgen-independent prostate cancer. Cell.

[bb0410] Wang Y., Nangia-Makker P., Tait L., Balan V., Hogan V., Pienta K.J., Raz A. (2009). Regulation of prostate cancer progression by galectin-3. Am. J. Pathol..

[bb0415] Wang D., Dafik L., Nolley R., Huang W., Wolfinger R.D., Wang L.X., Peehl D.M. (2013). Anti-oligomannose antibodies as potential serum biomarkers of aggressive prostate cancer. Drug Dev. Res..

[bb0420] Watanabe Y., Takeuchi K., Higa Onaga S., Sato M., Tsujita M., Abe M., Natsume R., Li M., Furuichi T., Saeki M., Izumikawa T., Hasegawa A., Yokoyama M., Ikegawa S., Sakimura K., Amizuka N., Kitagawa H., Igarashi M. (2010). Chondroitin sulfate *N*-acetylgalactosaminyltransferase-1 is required for normal cartilage development. Biochem. J..

[bb0425] Wellen K.E., Lu C., Mancuso A., Lemons J.M., Ryczko M., Dennis J.W., Rabinowitz J.D., Coller H.A., Thompson C.B. (2010). The hexosamine biosynthetic pathway couples growth factor-induced glutamine uptake to glucose metabolism. Genes Dev..

[bb0430] Wu D., Zhang C., Shen Y., Nephew K.P., Wang Q. (2011). Androgen receptor-driven chromatin looping in prostate cancer. Trends Endocrinol. Metab..

[bb0435] Wu X., Daniels G., Lee P., Monaco M.E. (2014). Lipid metabolism in prostate cancer. Am. J. Clin. Exp. Urol..

[bb0440] Yoneyama T., Ohyama C., Hatakeyama S., Narita S., Habuchi T., Koie T., Mori K., Hidari K.I., Yamaguchi M., Suzuki T., Tobisawa Y. (2014). Measurement of aberrant glycosylation of prostate specific antigen can improve specificity in early detection of prostate cancer. Biochem. Biophys. Res. Commun..

[bb0445] Young M.D., Wakefield M.J., Smyth G.K., Oshlack A. (2010). Gene ontology analysis for RNA-seq: accounting for selection bias. Genome Biol..

