# Supplementary material for: Glycosylation is an Androgen-Regulated Process Essential for Prostate Cancer Cell Viability
Source: eBioMedicine. 2016 Apr 20;8:103–16. doi: 10.1016/j.ebiom.2016.04.018 (PMC4919605; doi:10.1016/j.ebiom.2016.04.018)

Supplementary Figure 1

Validation of glycosylation enzyme antibody specificity by siRNA mediated protein depletion

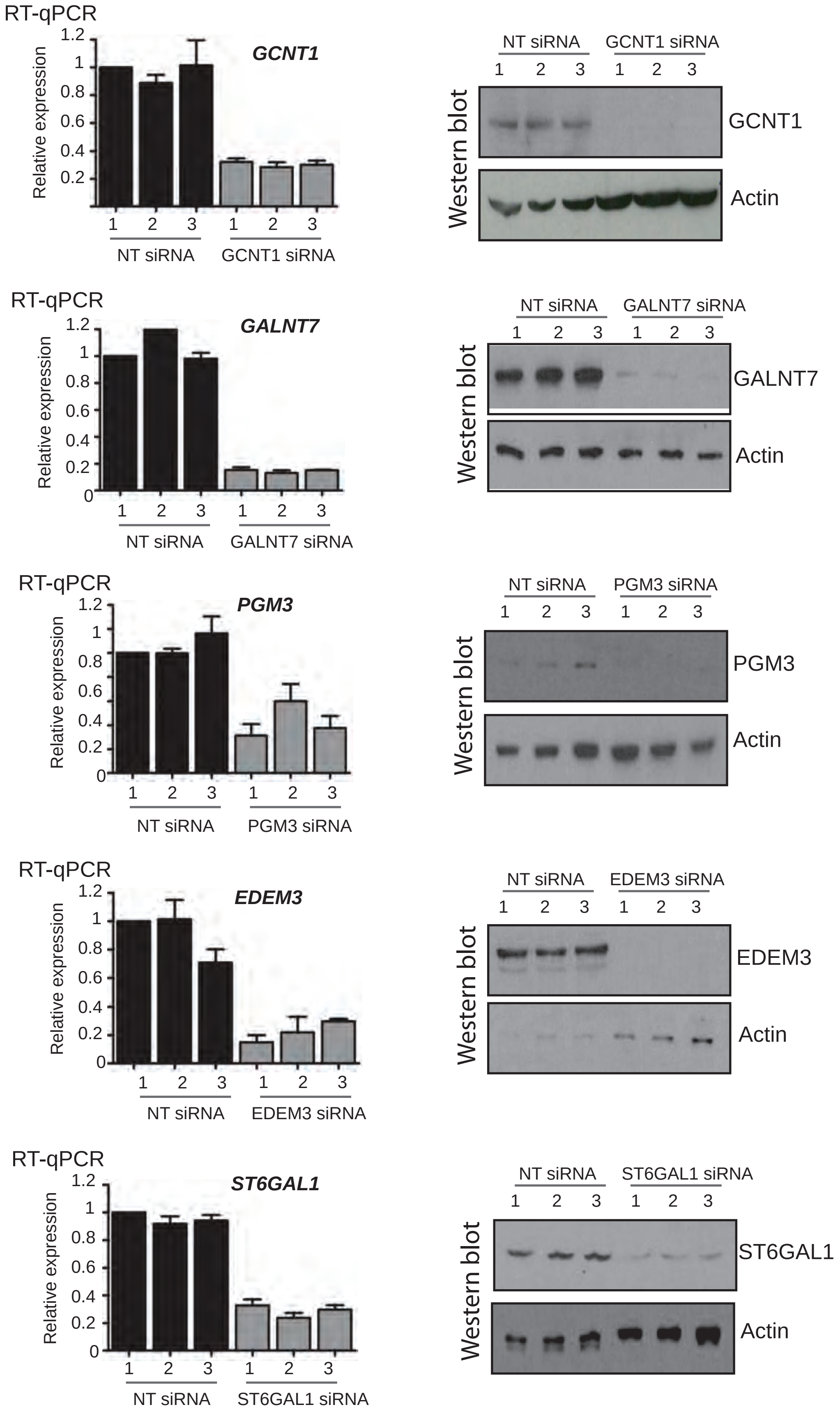

Supplementary Figure 2a

Triplicate RNA Sequencing data from LNCaP cells grown with or without androgens (R1881)

i) Expression level distribution plot

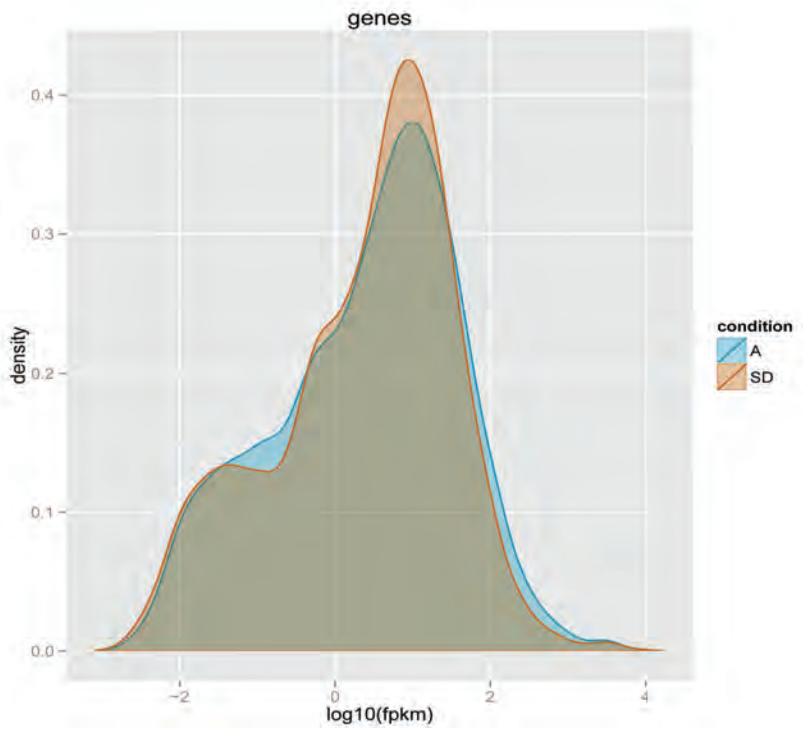

ii) Expression scatter plot

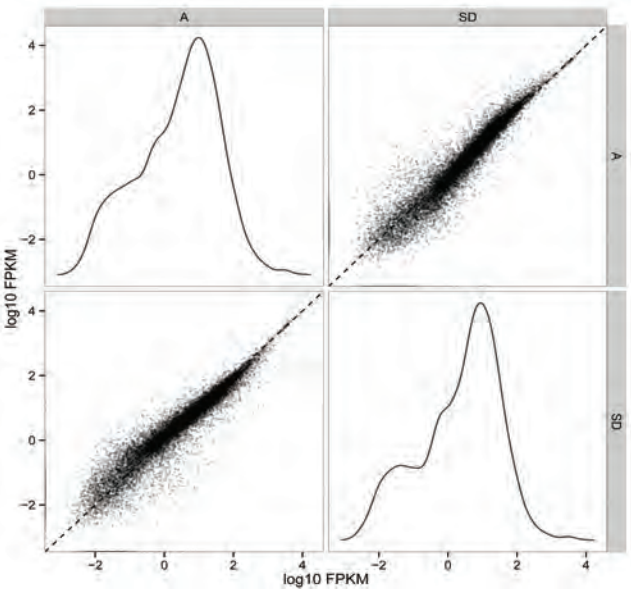

ii) Expression volcano plot

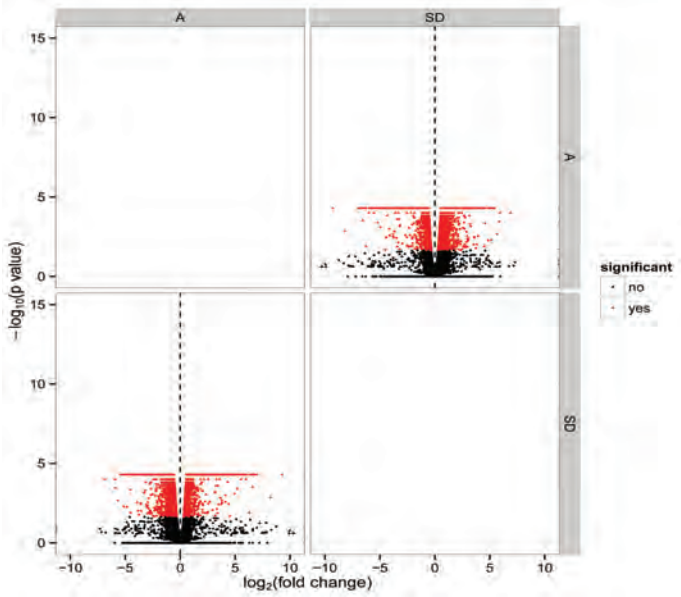

Supplementary Figure 2a

RNA Sequencing data from 7 prostate cancer patients pre- and post ADT

i) Expression level distribution plot

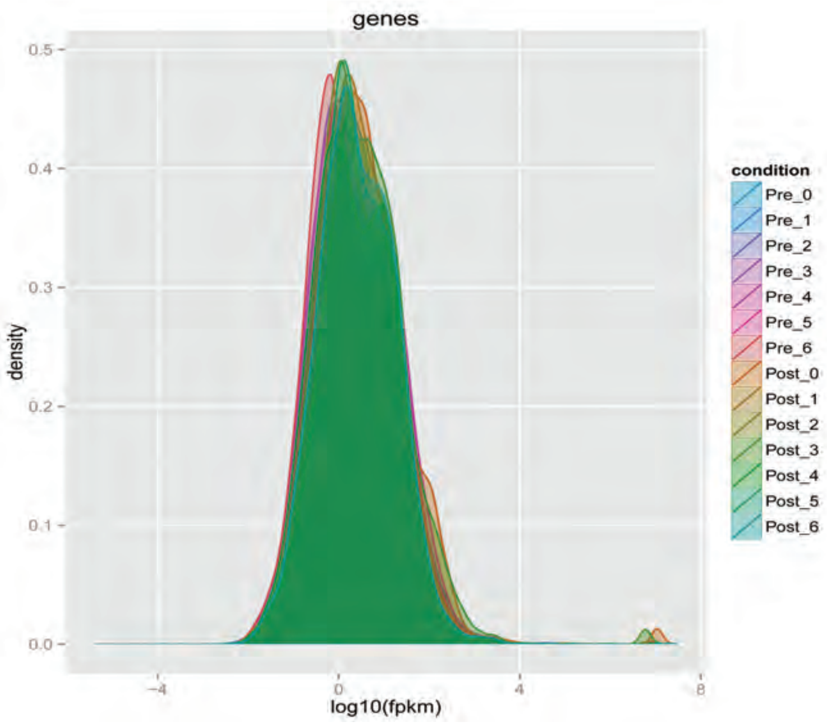

ii) Expression scatter plot

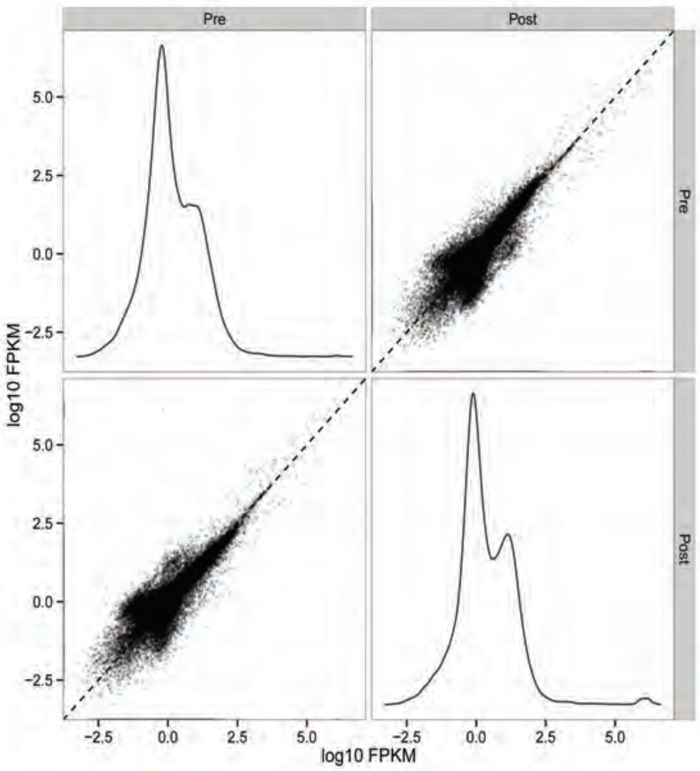

ii) Expression volcano plot

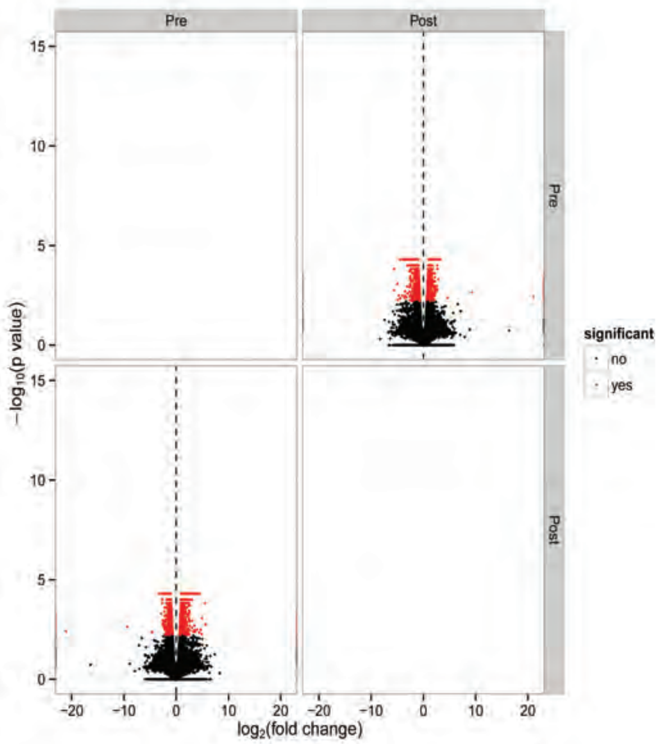

Supplementary Figure 3

a) LNCaP cells treated with different concentrations of R1881

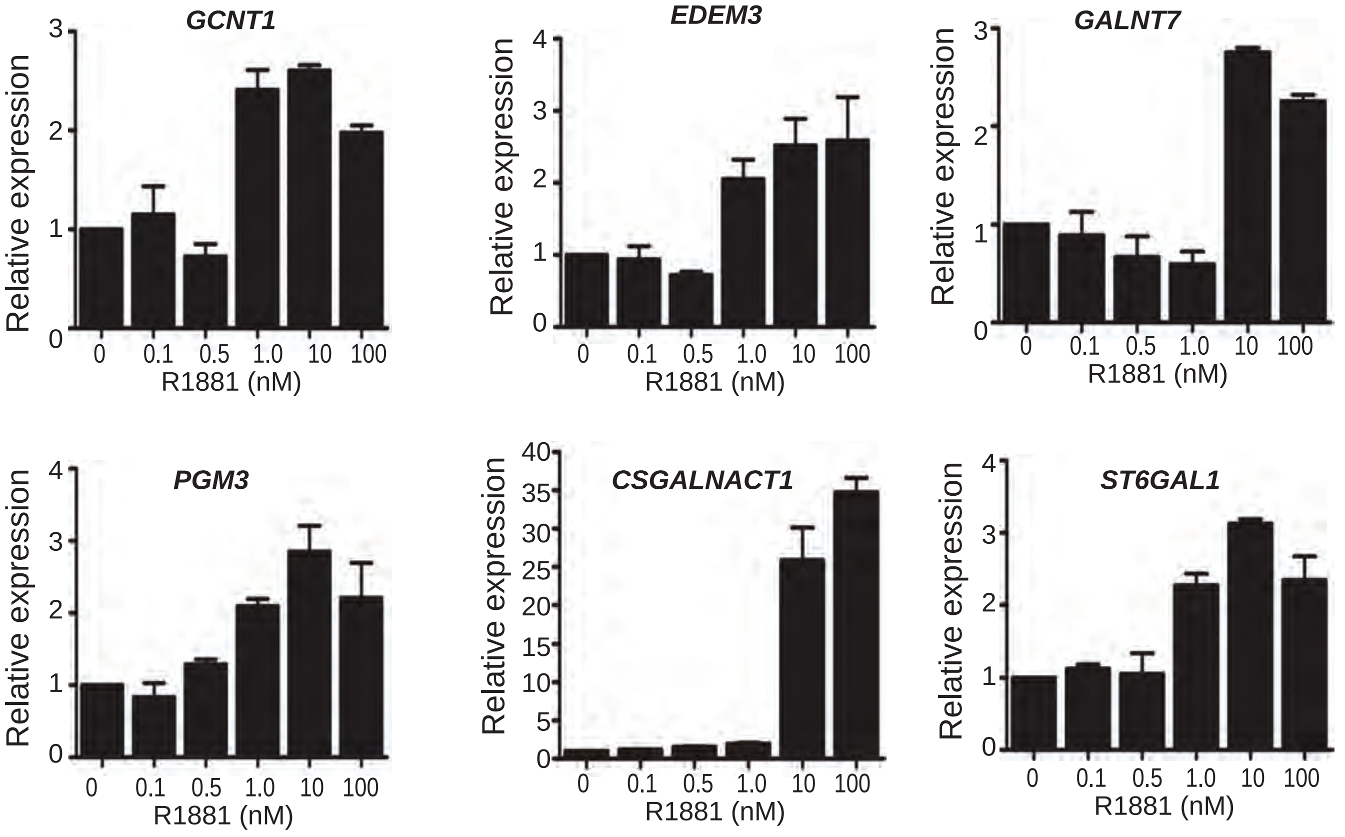

b) LNCaP cells treated with the androgen receptor antagonist Casodex

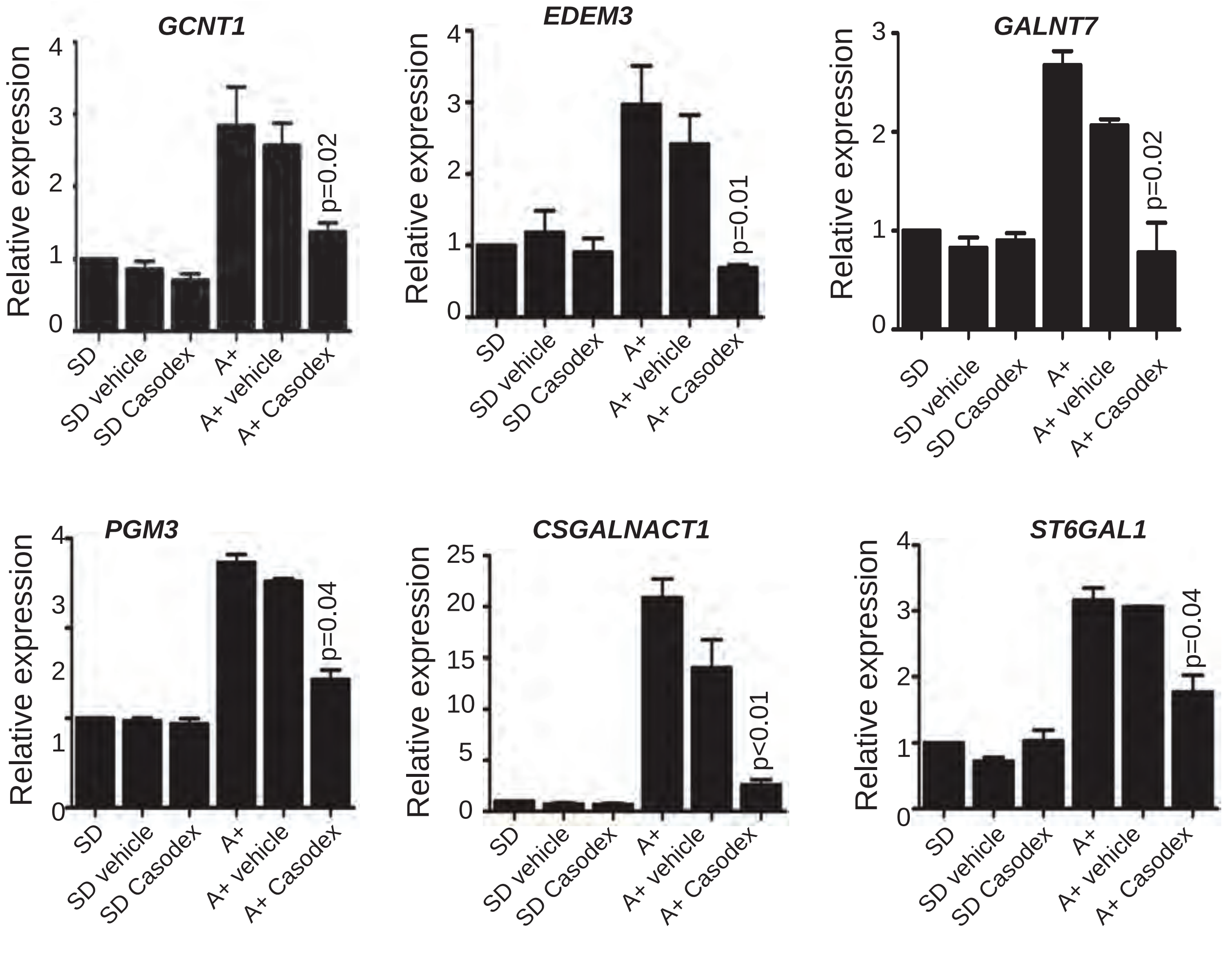

Supplementary Figure 4a,b

Induction of glycosylation enzyme genes glycans by androgens in VCaP cells

a) Real-time PCR analysis of glycosylation enzymes in VCaP cells.
Cells were treated with 10nM R1881 for 24 hours (A+) or without (steroid deplete, SD)

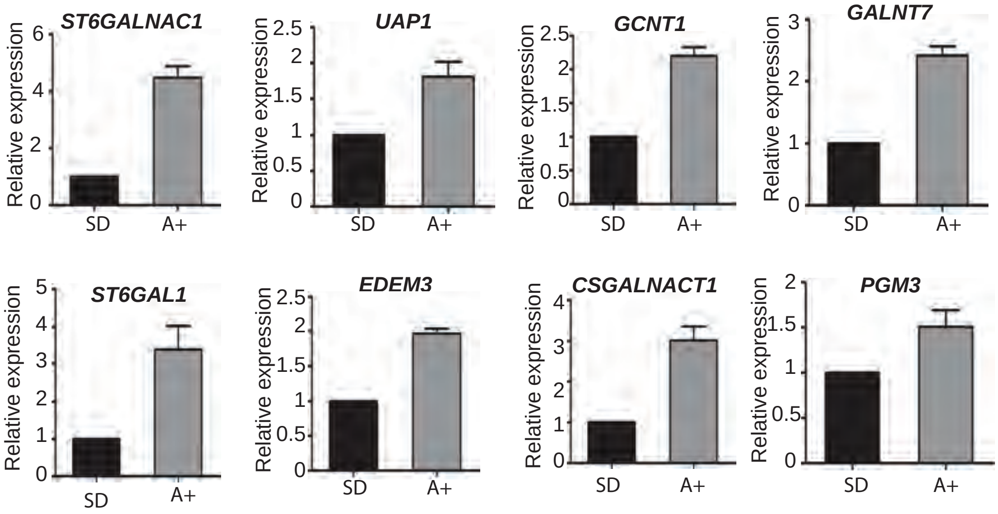

b) Analysis of lectin binding in VCaP cells
Cells were grown with or without 10nM R1881 for 72 hours prior to fixation

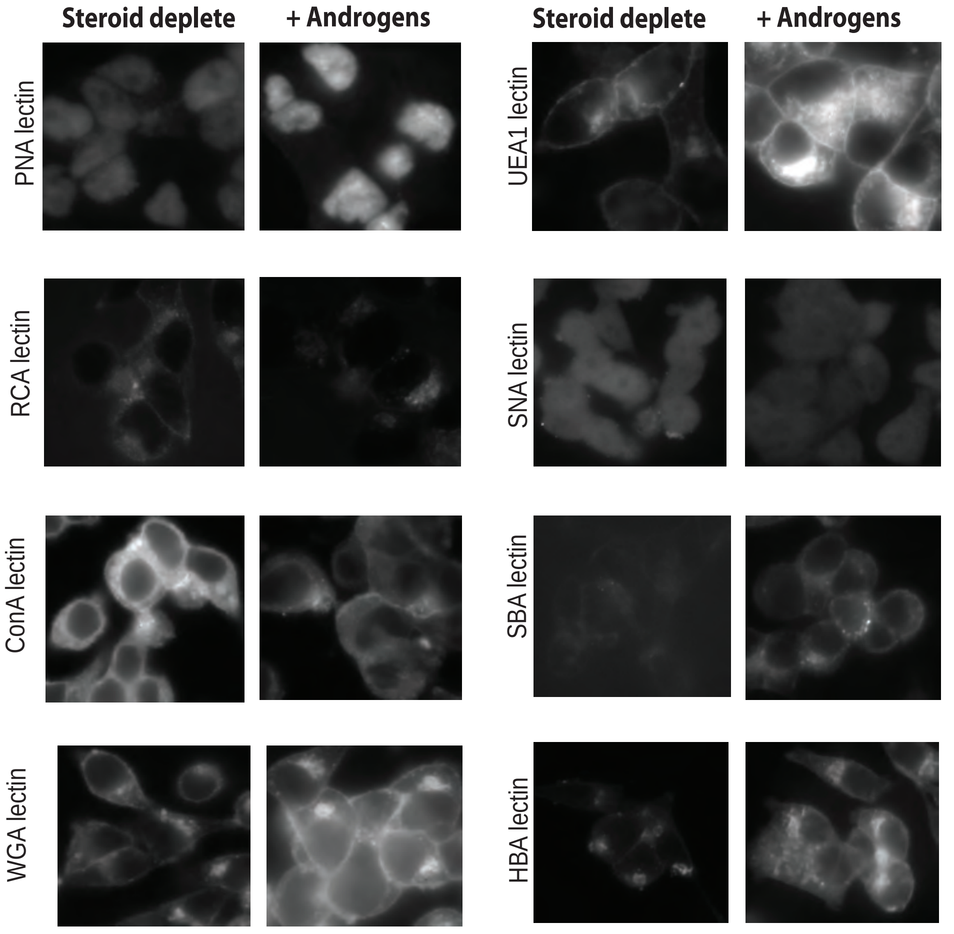

Supplementary Figure 4c

Induction of cancer-associated antigens by androgens in VCaP cells

VCaP cells were treated with or without androgens (10nM R1881) for 72 hours prior to fixation

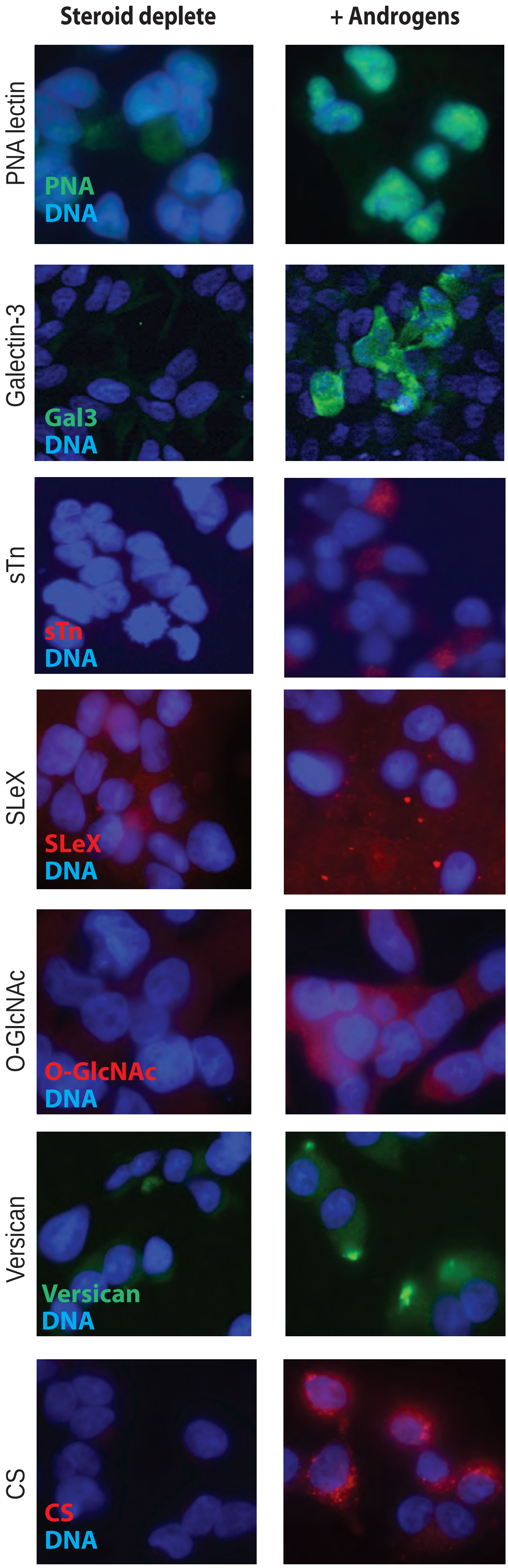

Supplementary Figure 5

Cell viability assay after siRNA mediated depletion of glycosylation enzymes in LNCaP cells

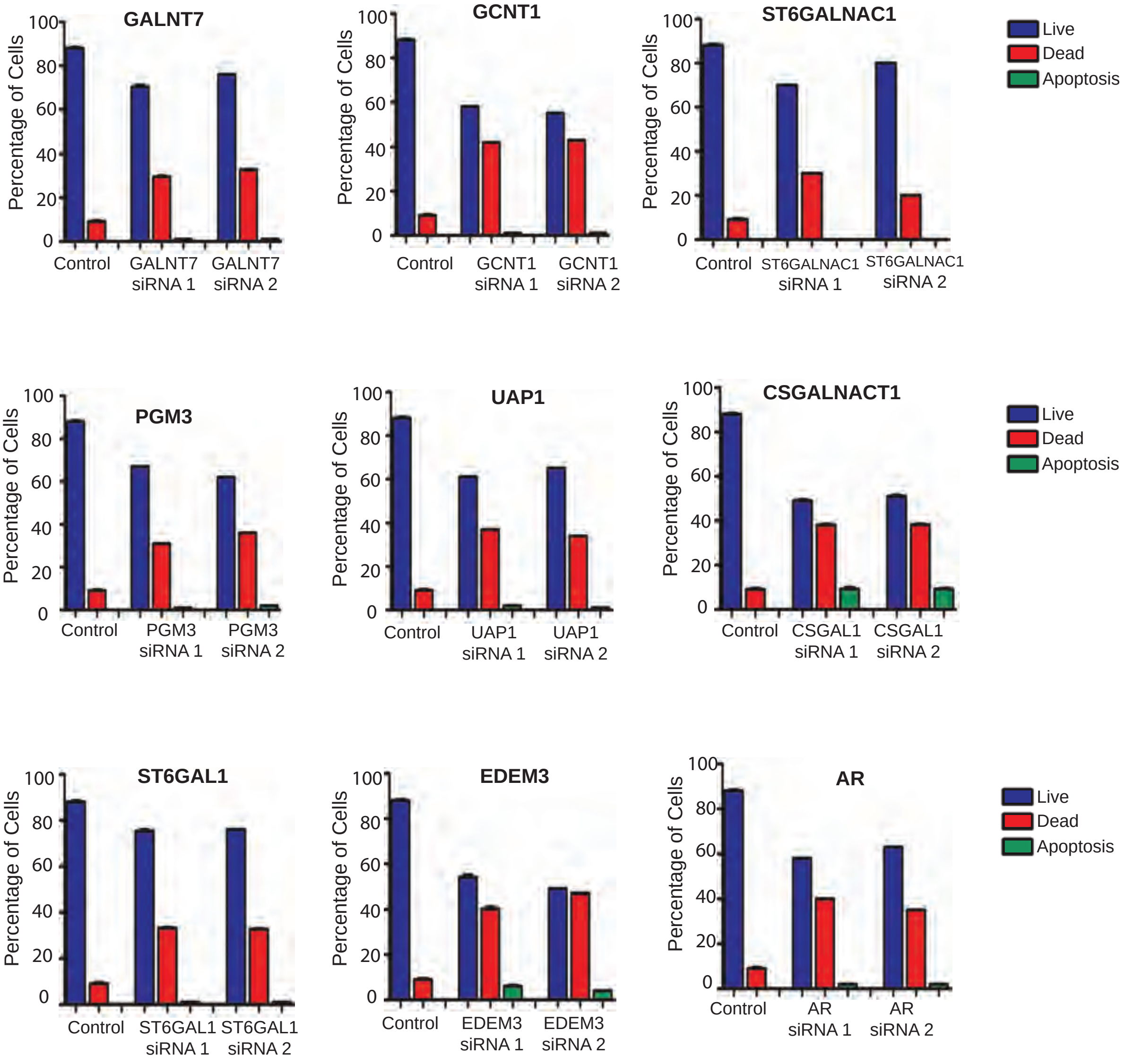

Supplementary Figure 6

siRNA mediated depletion of glycosylation enzymes in CWR22 RV1 cells

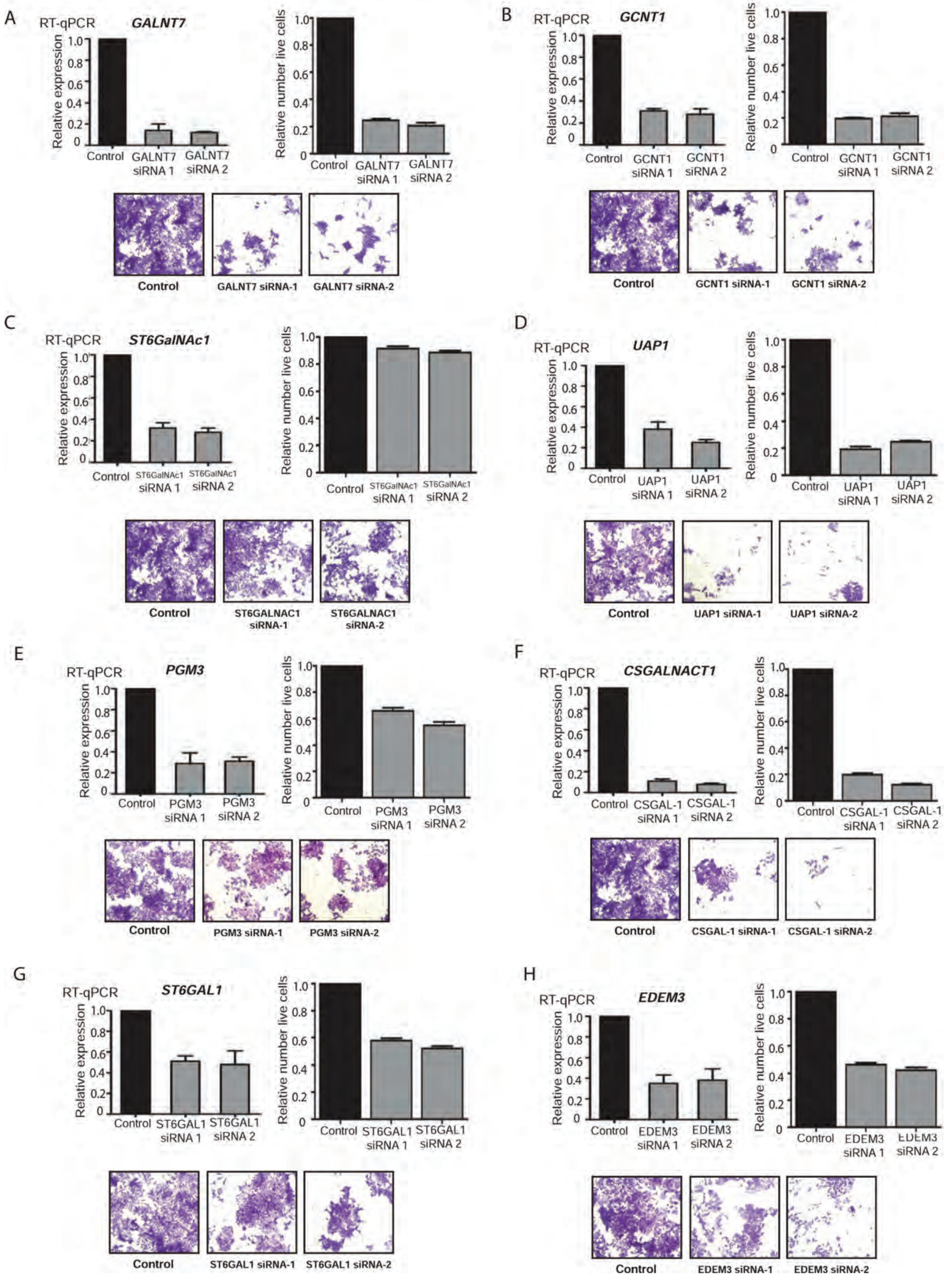

Supplement: Supplementary file 1 — Supplementary figures. [file mmc1.pdf]
